# The sense of safety theoretical framework: a trauma-informed and healing-oriented approach for whole person care

**DOI:** 10.3389/fpsyg.2024.1441493

**Published:** 2025-01-14

**Authors:** Johanna M. Lynch, Kurt C. Stange, Christopher Dowrick, Linn Getz, Pamela J. Meredith, Mieke L. Van Driel, Meredith G. Harris, Kate Tillack, Caley Tapp

**Affiliations:** ^1^General Practice Clinical Unit, Faculty of Medicine, The University of Queensland, Brisbane, QLD, Australia; ^2^Center for Community Health Integration and Departments of Family Medicine and Community Health, Population and Quantitative Health Sciences, and Sociology, Case Western Reserve University, Cleveland, OH, United States; ^3^Primary Medical Care, The Institute of Population Health, University of Liverpool, Liverpool, United Kingdom; ^4^General Practice Research Unit, Department of Public Health and Nursing, Faculty of Medicine and Health Sciences, Norwegian University of Science and Technology (NTNU), Trondheim, Norway; ^5^Occupational Therapy, School of Health, University of the Sunshine Coast, Sunshine Coast, QLD, Australia; ^6^School of Population Health, Faculty of Medicine, The University of Queensland, Brisbane, QLD, Australia; ^7^Queensland Centre for Mental Health Research, The Park Centre for Mental Health, Brisbane, QLD, Australia

**Keywords:** sense of safety, distress, whole person care, transdisciplinary, trauma-informed, healing-oriented, embodied, primary care

## Abstract

**Objectives:**

This research describes four aspects of the development of the Sense of Safety Theoretical Framework for whole person care: exploring the meaning of the phrase “sense of safety”—the whole person *language*; the range of human experience that impacts sense of safety—whole person *scope*; the dynamics that build sense of safety—the healing *goals*; and the personal and cross-disciplinary trauma-informed practitioner *skills and attitudes* that facilitate sense of safety.

**Methods:**

This qualitative participatory study was conducted in two phases. Researchers iteratively explored the concept of sense of safety using focus groups and semi-structured interviews. Overarching research questions were: “Does the transdisciplinary concept of Sense of Safety make sense as an approach to the whole person in distress?”; “How do participants describe the meaning of the phrase “sense of safety”?”; “What does a person experience when they feel safe?” and “What can practitioners do to facilitate a sense of safety?” Phase One involved rural and urban family doctors, mental health clinicians across multiple disciplines, people with lived experience of mental distress, and Indigenous Australian academics. Phase Two widened the scope of disciplines involved to iteratively reflect on their clinical and personal experience with “sense of safety” and included international family doctors, physiotherapists, occupational therapists, social workers, teachers, multidisciplinary rural clinicians and multidisciplinary clinicians with a lived experience of physical trauma, grief, and severe mental illness.

**Results:**

The everyday *language* “sense of safety” was found to describe a whole person experience that integrates awareness of self, others, and context. The *scope* of human experience that impacts sensed safety was found to include seven domains: Environment, Social Climate, Relationships, Body, Inner Experience, Sense of Self and Spirit/Meaning (Whole Person Domains). Five dynamic healing *goals* were identified that build sense of safety: Broad Awareness; Calm Sense-Making; Respectful Connection; Capable Engagement; and Owning Yourself (Sense of Safety Dynamics). Five practitioner *skills and attitudes* that facilitate sense of safety were named: Valuing the Whole Picture; Holding Story Safely; Being with You; Learning Together; and Validating Dignity (Sense of Safety Practitioner Skills).

**Conclusion:**

The Sense of Safety Theoretical Framework developed in this study focusses on an experience that is a fundamental prerequisite of health. Sense of safety is affected by, and influences, life story, relationships, meaning, sense of self, and – physical health: the whole person. The language “sense of safety” communicates an integrative experience that can help clinicians to see the whole person and describe a cross-disciplinary goal of care. The Whole Person Domains clarify the scope of care required, while the Sense of Safety Dynamics offer practical processes of care. The Sense of Safety Practitioner Skills describe trauma-informed skills and attitudes that facilitate a sense of safety. Each of these parts of the Sense of Safety Theoretical Framework translate practitioner, lived experience, and First Nations wisdom and a wide existing transdisciplinary literature into a framework and language ready for practice. Assessing and building sense of safety prioritizes a healing-oriented and trauma-informed approach. The Sense of Safety Theoretical Framework facilitates a paradigm shift that towards integrating sensation, subjective experience, physiology, and social determinants into everyday quality care in health, education and public policy.

## Introduction

Sensing we are safe matters where we live, learn, work, and where we receive care. When people feel safe, they are healthier; their bodies and minds are calmer, they sleep better, form relationships more easily, see things more clearly, and create more freely (Kark and Carmeli, 2009; Lynch, 2021; Perogamvros et al., 2020; Porges, 2011). Feeling safe is vital for resting, learning, growing, belonging, and healing (Burke-Harris, 2018; Maunder and Hunter, 2001). People have an overarching need to feel safe that Maslow called a “metaneed” (Maslow, 1954, p. 8). He said humans are “safety seeking” (Maslow, 1954, p. 1818). Seeking a sense of safety can drive behavior (Hunter and Maunder, 2001), shift physiological feedback systems (Juster et al., 2011), impact social connection (Porges and Carter, 2017), influence therapeutic relationship (Geller and Porges, 2014), and contribute to overall health. Although there is a growing understanding of the importance of sensed safety to health in psychotherapeutic, trauma and violence informed, attachment-informed, molecular stress science, and other fields like the built environment, these fields of research often remain siloed. The Sense of Safety Theoretical Framework uses a whole person lens to integrate many smaller parts of knowledge and ways of knowing in a way that gathers these fields together to advance healing and health beyond current practice.

The human organism responds to threat in an integrated way. The body does not differentiate between objective physical threats and subjective social or relational threats (Fleshner and Laudenslager, 2004). Alarm is a whole person experience—triggered by sensing both physical and social pain (Eisenberger and Lieberman, 2004). From immune processes to social assessment of facial expressions and the multilayered stress response system, people constantly assess threat and their capacity to respond to it (Porges, 2004). Appraisal of threat can come from external (physical, chemical, thermal, microbial, relational, or structural threat) or internal (mitotic, autoimmune, sensory, intrapsychic, memorial, or even existential) processes (Arnsten, 2009; Lynch, 2019; Whiting et al., 2012). Selye, who coined the term “stress” (Selye, 1950) named the impact of “stressors” on the whole person’s capacity to adapt (Selye, 1950; Selye, 1956). Modern stress researchers confirm the impact of both psychological and physical threat on the body (Delpierre et al., 2016; Tomasdottir et al., 2015) and note the way that the neurological, cellular, immune, endocrine, and social engagement systems become vigilant and ready for action (Fagundes et al., 2013; Geisler et al., 2013; Shalev et al., 2013). The Generalised Unsafety Theory of Stress describes chronic default physiological stress responses including autonomic arousal that contribute to disease in response to “largely unconsciously perceived *unsafety*” (Brosschot et al., 2018). “Unsafety” (or loss or lack of sense of safety) can be experienced even in the absence of overt stressors. (Brosschot et al., 2018) and could unravel many medically unexplained symptoms or complex social influences. Chronic loss of sense of safety becomes encoded as multisystem dysregulation, or “allostatic overload” (Juster et al., 2010) that impacts health and life expectancy (Lynch, 2021; Lynch and Kirkengen, 2019; Brosschot et al., 2018; Juster et al., 2011). This has wide implications for health and public policy.

Not all unsettling experiences cause loss of sense of safety—as Selye noted in his term “eustress” (Selye, 1956). McEwen uses the terms “positive stress” to describe a personal challenge that results in a sense of mastery and esteem, and the term “tolerable stress” to describe adverse life events buffered by supportive relationships that lead to coping and recovery (McEwen and Karatsoreos, 2020). Long term impacts of what is called “toxic stress” only come from threat that is appraised as overwhelming the individual’s capacity to adapt (Van Praag et al., 2004) in the absence of supportive relationships (Shonkoff et al., 2014). Sense of safety is impacted by *threat appraisal* (threat detection) systems (Teicher et al., 2016), including perception, attention, arousal, anticipation, and sensation; *coping appraisal* systems (sense of capacity to respond to the threat; Matthieu and Ivanoff, 2006; Brosschot et al., 2018); *perception of social support* (Timperio et al., 2015); and *meaning-making* systems (sense of coherence that the world makes sense; Antonovsky and Sagy, 1986). The concept of sense of safety therefore naturally draws attention to personal, communal, and meaning-making strengths and resources for coping and growth. Awareness of the importance of sensing safety can mean that defenses, such as obsessions, addictions, avoidance, attempts at mastery, or health risk behaviors (Maunder and Hunter, 2016), can be understood as reasonable, meaningful, and purposeful attempts to sense safety in response to real, perceived, or anticipated danger (Sampson, 1990). It also explains how perceptions of supportive relationships are a vital part of health (Hunter and Maunder, 2001), and how shattered assumptions about life (Janoff-Bulman, 1985), hopelessness and meaninglessness (Newcomb and Harlow, 1986), uncertainty (Brosschot et al., 2018), or loss of cultural safety (Curtis et al., 2019) impact whole person health and wellbeing.

The Sense of Safety Theoretical Framework described in this paper integrates a number of well described theories—including Attachment Theory (Bowlby, 1984; Maunder and Hunter, 2001; Bowlby, 1979), Maslow’s Theory of Motivation (Maslow, 1943), Allostatic Load Theory (McEwen, 2007; Lupien et al., 2009), Polyvagal Theory (Porges, 2011), Social Safety Theory (Slavich, 2020), Generalized Unsafety Theory of Stress (Brosschot et al., 2018), Theory of Human Security (Blatz, 1973) and Interpersonal Theory (Sullivan, 1953). Each of these theories, although far reaching in themselves, have practical limitations when seeking to apply them within the context of an integrated whole person approach to health, education, and public policy.

The Sense of Safety Theoretical Framework is built on generalist philosophy that highly values practical approaches that attend to both the biology and biography of each person as part of healing (Lynch et al., 2021; Lynch et al., 2020b; Stange, 2009; Scott et al., 2008). As outlined below, it also integrates evidence from psychotherapy, and fields that explore experiences that could be described as a “loss of sense of safety” or “unsafety,” including trauma, domestic violence, loneliness, social rejection. The Sense of Safety Theoretical Framework seeks to integrate across these diverse fields of research to offer practical trauma-informed, healing-oriented, whole person approaches to community distress.

Attachment Theory in its focus on safe relationships provides a key element of the theoretical underpinning of the Sense of Safety Theoretical Framework. This body of literature includes applications in neurodevelopment (Schore, 2001; Sullivan, 2003), sense of self (Mikulincer, 1995), emotion regulation (Padykula and Conklin, 2010), chronic pain (Smith et al., 2018), cellular stress (Murdock et al., 2018), romantic attachment (Mikulincer and Shaver, 2007), social networks (Gillath et al., 2017), parenting (Powell et al., 2013), therapeutic relationship (Maunder and Hunter, 2016; McWilliams, 2018), and even spiritual relationships (Vehling et al., 2019; Scheffold et al., 2019). But attachment cannot explain all impacts on sense of safety and stops short of conceptualization of wider social determinants or cultural impacts on health. It does use a similar term for the phenomenon of sensed safety—“felt security” (Allen and Manning, 2007, p. 30) but does not have an overarching theoretical approach to the whole person. This term is also not as everyday as the ordinary English phrase “sense of safety” and not as embodied or biologically relevant as the term “sense.”

Other fields of literature that explore relational safety and loss of safe relationships (including with ourself) also add depth to our understanding of the lifelong impact of attachment. Each of these fields underscore the importance of relationships and social connections as part of the Sense of Safety Theoretical Framework. Early childhood experiences of safety facilitate affect regulation, neural networks and connectivity, integration of sensory and narrative information, and the formation of a stable sense of self (Schore, 2003; Mikulincer, 1995; Lupien et al., 2009). Attuned responsive relationships co-regulate emotion (Butler and Randall, 2013), signal safety at a neurological level (Eisenberger et al., 2011). Attachment is described as a “safety regulating system” (Allen and Manning, 2007, p. 23) comprising differing caregiving and care-receiving systems. These include attuned relational experiences of both *safe haven* (providing soothing comfort and refuge) and *secure base* (encouraging capacity to engage with the world and take appropriate risks to step out, explore, learn and grow) (Kerns et al., 2015). Fear modulation through relationship has been identified as a significant “hidden regulator” (Coan et al., 2006, p. 1038) of neurodevelopment and physiological health (Caballero et al., 2023). Loss of lack of sense of safety is a part of loneliness (Hawkley and Cacioppo, 2010), social exclusion (Baumeister et al., 2007), social rejection (Slavich et al., 2010), social pain (Eisenberger et al., 2011), betrayal (Freyd et al., 2005), and bullying (Lereya et al., 2015). Internal relationships with self can also impact sense of safety. Self-loathing (Dorahy et al., 2015; Gruenewald et al., 2004), self-criticism (Priel and Shahar, 2000), or other forms of empathic failure towards the self (Neimeyer and Jordan, 2002) are a kind of inner estrangement (Stamenov, 2003) that is threatening (Frewen et al., 2015). Unnoticed loss of lack of sense of safety or “unsafety” (Brosschot et al., 2018) is an underlying unaddressed factor in many chronic and complex presentations to global health and social services systems.

Support for the Sense of Safety Theoretical Framework is also found in literature regarding trauma, with goals of safety and sanctuary built into models of trauma care (Bloom and Farragher, 2013; Brand, 2001; Herman, 2015) and guidelines for trauma-informed care (Kezelman and Stavropoulos, 2012). Trauma researchers have identified the broad impacts of chronic threat—altering perception and trust in relationships to self, others, meaning, consciousness and connection to body (Courtois, 2004). They describe the therapeutic goal of restoring a “visceral sense of control and safety” (Van Der Kolk, 2014, p. 31) and “sense of safety and stability” (Enns et al., 1998, p. 248) that increases affect regulation, sense of mastery, capacity to cope, and strengthened social relationships. They describe the loss of capacity to “feel safe with other humans, or even with themselves” (Frewen et al., 2015, p. xiv) caused by flashbacks, hyperarousal, and avoidance. They also note the importance of perceiving safety as an active process (Cai et al., 2014) marked by the physical signs of sense of safety in posture, breathing, prosody, capacity to express emotion, and self-acceptance (Rappoport, 1997, p. 253). Research into trauma and neglect has established the dose-dependent impact of trauma (a kind of loss of sense of safety) on physical, emotional and social health—including life expectancy (Felitti et al., 1998). The trauma (McEwen, 2002; Courtois and Ford, 2014; Lanius et al., 2001), childhood maltreatment (Teicher et al., 2003; Scott et al., 2023), and adverse childhood experiences literature (Felitti et al., 2019) have an expanding understanding of the impact of adversity on long term physical health, perception, relationships, sense of self, meaning making, and hope. However, trauma informed approaches are currently quite narrow in how they categorise what is traumatic, often assessing from the point of view of the practitioner without attending to the internal experience of the person who has been wounded. Seeing trauma as a kind of loss of sense of safety could shift attention towards the phenomenon of sensed safety. This could widen practitioner awareness to notice hidden processes that traumatize (for example neglect, disenfranchised grief, or forms of coercive control). It can also prioritize a healing-orientation that notices strengths and resources that build sense of safety. This will increase clinical sensitivity to unnoticed trauma, move attention to “what next,” enrich our understanding of the many ways that lived experience impacts each person, and raise awareness of the ways that sensing that we are safe offers healing.

The trauma and stress fields of research have also developed theories that are relevant to understanding sensed safety and its impact on biology. The toxic stress (Shonkoff et al., 2012) and allostatic load (Juster et al., 2010) literature and Polyvagal Theory (Porges, 2011) all implicitly address the physiological impact of loss of sense of safety. They confirm the impact of lived experience and relationships on physical health that has been explored in psychophysiology (Berntson et al., 2007), psychoneuroimmunology (Fleshner and Laudenslager, 2004), interpersonal biology (Siegel, 2001), and somatosensory (Kross et al., 2011) fields of research. Porges has termed the phrase “neuroception” to describe the process of sensing or appraising threat that is in effect sensed loss of safety (Porges, 2004). This body of work has illuminated the importance of physical experiences of safety on lifelong health. It includes the work of Gilbert (Gilbert, 1993) and Slavich (Slavich, 2020) who outline the far-reaching impact and theoretical links to evolutionary biology of feeling safe socially. These bodies of literature add theoretical foundation to the Sense of Safety Theoretical Framework but do not include all aspects of the whole person, or offer a practical language, a defined scope, shared goals of care, or overarching practitioner skills ready for translation into practice. The interconnectedness of social and physical experiences of sensed safety impact immunology, neurobiology, cellular biology and overall health and therefore must be considered in health, education, social services, and public health policy.

The Sense of Safety Theoretical Framework also includes awareness of contextual security as part of the whole. This includes social determinants of health (Marmot, 2005), living conditions such as housing security (Thurston et al., 2013), crowding, and noise (Sayin et al., 2015), psychological safety at work (Kark and Carmeli, 2009), and cultural safety (Lavrencic et al., 2021). The built environment literature does actually use the term “sense of safety” as a measure of wellbeing. Although this confirms the relevance of the phenomenon of sensed safety in the environment around the person, the use of this term in this literature is mostly limited to correlation between sense of safety and solidarity with neighbors and green and blue spaces near where you live (Kuo et al., 1998; White et al., 2010). Environmental research also confirms the relevance of water, food, political, and international security to personal experiences of safety (Cook and Bakker, 2012; Gleick, 1993; Pinstrup-Andersen, 2009). Processes of injustice, incarceration corruption, migration, and racism also impact safety and health across communities (Chao et al., 2014; Levy and Sidel, 2013). While sense of safety is implicit across this broad literature, the links to wellbeing of the whole person have not been explicitly examined or drawn together ready for use in practice, research or public policy. This has limited clinical and community-wide application of this important body of research.

Senses allow us to gain information about the world (Marks, 2014, p. 40) and they have a purpose—to protect our physical and moral *integrity*, our sense of *connection* to others, and our sense of *coherence* that the world “makes sense” (Lynch, 2021). Paying attention to sensation can widen awareness to notice previously disregarded personal, communal, environmental, and even historical and intergenerational causes of threat that impact wellbeing. It can also shift attention towards therapeutic or healing goals of care: to build sense of safety. As we consider moving language and awareness from “safety” to “sense of safety” in frameworks of care it is important to notice the inherent paradigm shift. As soon as we acknowledge “sense of” we are embracing the value of subjective sensation and meaning-making that is inside the person, alongside other more objective approaches to understanding. Sensing safety is an integrative response to both external and internal sources of threat to (or resources for) safety. The Sense of Safety Theoretical Framework includes both observed safety and the more wholistic and complex phenomenon of sensed safety. This framework proactively removes artificial distinctions between observed or experienced threat or capacity across the whole person. Maslow’s Theory of Motivation offers strong theoretical foundation for this work and does attend to inner motivation. There is attention to needs across the whole person in physiology, environmental safety, love and belonging, esteem, self-actualization, and self transcendence (Maslow, 1954; Koltko-Rivera, 2006). However, Maslow does not acknowledge subjective sensed safety across the whole person that this Sense of Safety Theoretical Framework addresses.

A World Health Organization collaborative definition of safety named two dimensions of safety—an objective external safety (what they denoted “real safety”) and an internal perception of safety (named “perceived safety”; Nilsen et al., 2004)—resulting in a definition of safety: “a state or situation devoid of physical, material or moral threats, which must lead to a perception of being sheltered from danger” (Maurice et al., 1997, p. 181).This perception of feeling sheltered from danger has real impact on the whole person—it impacts their relationships, their meaning, their levels of physiological arousal and health. The Sense of Safety Theoretical Framework includes objective external safety, internal perception of safety, and intuitive embodied sensations of safety, and names them all “real.” Although perception and sensation are interconnected and both use cognitive and sensory appraisal, the term “sense of safety” is used in this framework (rather than ‘perceived safety’) because it is everyday English and helps practitioner and patient to stay attuned to the role of the sensory body in appraisal. This subjective experience of sensing safety is implicit in many approaches to public health, health and safety quality control processes, clinical care, and approaches to clinician wellbeing. The Sense of Safety Theoretical Framework will make it explicit and is designed for use in each of these settings.

Clinical awareness of each person’s sense of safety may be a window into understanding the impact of lived experience on health at multiple levels across the internal subjective and external objective appraisal and coping systems; social support and meaning-making systems; and care receiving and care giving systems. The Sense of Safety Theoretical Framework offers a coherent whole person way to understand and care for distress and prevent illness and disease. It translates a trauma-informed and healing-oriented approach to whole person care into everyday health, education, and social services practice, research and public policy. “Sense of safety” is an ordinary English phrase that holds promise as an accessible way to assess distress and define treatment goals. It is relevant to physiological, relational, spiritual, and psychological health. It could be useful across the disciplines if we had a shared understanding of its meaning and how to build it into therapeutic processes and public policy.

This study sought to explore the meaning of the phrase “sense of safety” to family doctors, multidisciplinary mental health clinicians, Australian Indigenous academics, and people with a lived experience of mental illness and psychosocial distress. In a second phase, we further explored the concept of sense of safety with a broader international range of multidisciplinary practitioners (including some clinicians with a lived experience of physical trauma, bereavement and severe mental illness) seeking their insights on how clinicians already use awareness of sensed safety in practice. We sought to define the breadth of attention and practitioner skills and attitudes needed if both objective and subjective causes of threat (and resources for) safety were considered part of clinical assessment and treatment. We submitted our findings in iterative spirals to both participants and multidisciplinary academics for critique and review.

This study asked key research questions that explore the usefulness of the ordinary phrase “sense of safety” as a transdisciplinary approach to distress that defined breadth of whole person care and clarified healing goals. To prepare for translation into practice, both phases of research also sought to capture ways that practitioners already appraised sense of safety. Key research questions included: “Does the transdisciplinary concept of Sense of Safety make sense as an approach to the whole person in distress?”; “How do participants describe the meaning of the phrase ‘sense of safety’?”; “What does a person experience when they feel safe?”; “What helps a person to feel safe?”; “How do participants and an academic panel respond to the concept of ‘sense of safety’ and ‘What can practitioners do to facilitate a sense of safety?’” Fundamentally, this study sought to ask a broad question: how can we operationalize our theoretical understanding of the importance of sensed safety to health and wellbeing to provide clear frameworks and goals of care for practice across the disciplines?

## Materials and methods

### Study design

This participatory research was designed and delivered by a family doctor (JML) using transdisciplinary generalist methodology (Lynch et al., 2020a; McGregor, 2018). In two phases, this study utilized participatory and iterative methods to explore the meaning of the ordinary phrase “sense of safety,” to map causes of threat, to observe verbs describing movement towards “sense of safety,” to ask participants about their reactions to the concept of “sense of safety,” and to ask clinicians to imagine using the concept in practice. Phase One was supervised by a senior academic international multidisciplinary team of three: two family doctors (CFD, MvD) and an occupational therapist (PJM). Additional supervision from experienced family doctors (LOG, KCS) and independent review of the data (KT) was added in Phase Two.

Phase One of the research involved focus groups and semi-structured interviews with practitioners and patients as outlined in Table 1. Phase Two involved multidisciplinary focus groups that explored the concepts through their disciplinary lens. Phase One findings were critiqued by an international academic panel after an early iteration and prior to reporting back to participants.

**Table 1:** Summary of participants in Phase One and Two.

Participants in Phase One and Two of sense of safety study			
Phase One (*n* = 40)	Group members	*n*=	Code
Focus Group 1	Lived experience of mental illness	6	le
Semi-structured interview 1	Rural GP	1	gp
Semi-structured interview 2	Mental health nurse	1	mhc
Semi-structured interview 3	Rural GP	1	gp
Focus Group 2	Urban GPs	7	gp
Focus Group 3	Lived experience of mental illness	3	le
Focus Group 4	Multidisciplinary mental health clinicians	10	mhc
Focus Group 4	Multidisciplinary mental health clinicians	7	mhc
Semi-structured interview 4	Rural GP	1	gp
Semi-structured interview 5	Rural GP	1	gp
Semi-structured interview 6	Australian Indigenous Academic	1	Ia
Semi-structured interview 7	Australian Indigenous Academic	1	Ia
Phase Two (*n* = 76)	Group members	*n*=	code
Focus Group a	USA GPs (16) and Netherland GP (1)	17	gp1-17a
Focus Group b	Domestic and family violence social workers	7	dfv1-7b
Focus Group c	Australian GPs (7) and allied health primary health researchers (4)	11	gp1-7c and PC1-4c
Focus Group d	Australian GPs	7	gp 1-7d
Focus Group e	Mental health clinicians with lived experience of physical trauma, severe mental illness and bereavement	3	mhc1-3 e
Focus Group f	Norwegian GPs (7) and one obstetrician	7	gp 1-7f and o/g1f
Focus Group g	Domestic and family violence social workers	2	dfv1-2g
Focus Group h	Teacher—primary	1	Te1h
Focus Group i	Occupational therapists (3) and physiotherapists (6)	9	OT and P1-9i
Focus Group j	Teachers primary (1) and high school (4)	5	Te1-5j
Focus Group k	Rural allied health team—speech pathologist, counsellor, teachers(2), literacy workers (2), occupationa therapist	7	Ru1-7 k

Phase Two (*n* = 76)	Group members	*n*=	Code
Focus Group a	USA GPs (16) and Netherland GP (1)	17	gp1-17a
Focus Group b	Domestic and family violence social workers	7	dfv1-7b
Focus Group c	Australian GPs (7) and allied health primary health researchers (4)	11	gp1-7c and PC1-4c
Focus Group d	Australian GPs	7	gp 1-7d
Focus Group e	Mental health clinicians with lived experience of physical trauma, severe mental illness and bereavement	3	mhc1-3 e
Focus Group f	Norwegian GPs (7) and one obstetrician	7	gp 1-7f and o/g1f
Focus Group g	Domestic and family violence social workers	2	dfv1-2g
Focus Group h	Teacher—primary	1	Te1h
Focus Group i	Occupational therapists (3) and physiotherapists (6)	9	OT and P1-9i
Focus Group j	Teachers primary (1) and high school (4)	5	Te1-5j
Focus Group k	Rural allied health team—speech pathologist, counsellor, teachers(2), literacy workers (2), occupationa therapist	7	Ru1-7 k

In Phase Two three participants self-identified as Indigenous [USA (1) and Australian(2). Denotations in this document for each type of experience are: lived experience (le), Indigenous academic (Ia) family doctor (gp), obstetrician/gynaecologist (o/g), teacher (Te), physiotherapist (P), occupational therapists (OT), domestic violence support social workers (dfv), mental health clinicians (mhc), rural team (Ru), allied health primary health researchers (PC). Academic advisory panel members are denoted by an A ahead of the rest of the descriptor. Each focus group in Phase Two is denoted using the letters a–k].

### Participants

As outlined in Table 1, Phase One (*n* = 40) included people with a lived experience of severe mental illness (*n* = 9) who were well supported in a patient advocacy service; Australian academics with both lived and academic expertise in Aboriginal and Torres Strait Islander wellbeing (Ia) (*n* = 2); rural (*n* = 4) and urban (*n* = 7) family doctors (otherwise known as general practitioners) recruited through an Australian professional organization for clinicians interested in mental health; and mental health clinicians (mhc) (*n* = 18) including psychologists, occupational therapists, social workers, nurses, counsellors, and pastoral care workers who were recruited at mental health conferences and network meetings.

Phase Two (*n* = 76) included family doctors from the USA (*n* = 16), The Netherlands (*n* = 1), Norway (*n* = 7), and Australia (*n* = 13); a gynecologist (*n* = 1); allied health primary care researchers (*n* = 4); primary (*n* = 2) and high school teachers (*n* = 4); physiotherapists (*n* = 6); occupational therapists (*n* = 3); domestic violence support social workers (*n* = 9); generalist mental health clinicians (*n* = 3); and multidisciplinary rural allied health team (*n* = 7; speech pathologist, counsellor, two teachers, occupational therapist, and two literacy workers).

Academic advisory panel members in Phase One included clinical academics: two psychiatrists (from Canada and Australia), three family doctors (from UK, USA, and Norway), four psychologists (from New Zealand, USA, and Australia),one mental health nurse (from Australia), one social science researcher and a psychotherapist from Australia. They were purposively selected for international standing and expertise.

Participants consulted as part of this project were purposively selected across multiple disciplines as informants with personal or professional experience of managing complex distress in healthcare, education or the social services. Lived experience participants were recruited through internal advertising at a patient advocacy service. Indigenous academics were recruited based on their reputation through direct request. Family doctors and primary care researchers were recruited through Australian and international professional organizations and university departments. Teachers, physiotherapists, and occupational therapists were recruited through snowballing from researcher contacts, Multidisciplinary mental health clinicians were recruited through advertising in professional organizations and at a conference. Clear informed consent protocols were followed and access to emotional support was available. Participants did not receive any financial remuneration.

Denotations in this document for each type of experience are: lived experience (le), Indigenous academic (Ia) family doctor (gp), obstetrician/gynaecologist (o/g), teacher (Te), physiotherapist (P), occupational therapists (OT), domestic violence support social workers (dfv), mental health clinicians (mhc), rural team (Ru). Academic advisory panel members are denoted by an A ahead of the rest of the descriptor. Each focus group in Phase Two is denoted using the letters a-k (see Table 1 for more details).

### Ethics statement

Phase One ethical approval was provided by The University of Queensland School of Medicine Low Risk Ethical Review Committee (2017-SOMILRE-0191) with an amendment in 2018 (2018000392) to allow inclusion of iterative feedback from international consultation that year. Phase Two ethical approval was provided by the University of Queensland School of Medicine Low Risk Ethical Review Committee (2021/HE002268) All participants received verbal and written information on the aim of the research, information on pathways to emotional support, and details about how their data would be deidentified following interview to maintain confidentiality. All participants provided written informed consent.

### Positionality of authors

As qualitative research is influenced at many levels by the research team, we discuss here the members of our team. All have Caucasian heritage. Six of the eight member team are women and three are early career researchers. JML is an Australian family doctor, psychotherapist and skilled qualitative researcher with clinical expertise in trauma-informed care. KCS is an experienced American family doctor, primary care policy leader, and qualitative and quantitative researcher. CFD is an experienced British family doctor and qualitative mental health researcher. LOG is an experienced Norwegian family doctor with experience in multimorbidity and allostatic load research. PJM is an Australian occupational therapist with experience in attachment and chronic pain. MvD is an experienced Australian family doctor and primary care researcher. MGH is an experienced mental health services evaluation researcher. CT is a social psychology researcher with quantitative and qualitative research skills. KT is a psychologist with qualitative research skills. All authors have reflected on the Sense of Safety Theoretical Framework in light of their own clinical and/or personal experiences of distress.

### Phase one and two topic guides

Both phases used open questions, multiple ways to communicate (written and oral) and expert facilitation that enabled group member comfort and participation. In face-to-face groups (Phase One) post-it notes and written content were encouraged to enable quieter members to participate. In online focus groups (Phase Two), the chat box was used to communicate written content. In both phases, prior to description of the research topic, a couple of very open questions were used, and individual written responses were collected in order to seek people’s opinions before more discussion. Interview guides were used to facilitate some standardization of the data collection across different groups and individual interviews. Two researchers reviewed the questions used in each focus group and planned any iterative changes prior to the next group in both phases.

Phase One focused on three main questions with sub-questions that were tested iteratively: *What does the phrase “sense of safety*” *mean to you?* (each participant was asked to write their response prior to full introduction from researchers); *What threatens people?*; and *How do people sense that they are safe?* Sub-questions included: *What helps people sense that they are safe? What creates a sense of safety?*; *What kinds of things could a general practitioner (gp) do to help you have a sense of safety?* Later groups were also asked: *What aspects of a person’s life is it important to feel safe in?*; and *Which aspects of a person is it important not to miss when caring for distress?* GPs were also asked: *What do you think you already assess in distress?*.

The academic panel were asked: *Given your area of expertise, how does the Sense of Safety concept and approach make sense to you?*; *Are there any theoretical discrepancies evident to you in this concept?*; *Are there any areas of appraisal of distress that you think have been neglected in this concept?*; *Do you have any general comments about this concept’s overall validity and utility?*

Wider academic discussions when the Phase One findings were presented to psychiatrists in Canada, Norwegian GPs and a multidisciplinary group in the UK asked the questions: *What is your overall understanding of Sense of Safety?*; *What is your gut reaction to the concept of Sense of Safety?*; *Do you have any concerns about this concept?*

Phase Two also started with a written task of answering the questions: *What does the phrase “sense of safety*” *mean to you?* and *How do you currently look for patterns of threat in your patients/clients?* After a presentation of the findings from Phase One, participants were then asked to reflect on sensations and experiences of sensing safety, discuss that in dyads, and then come back to describe the experience. They were then invited to discuss questions designed to elicit their response to the concept of sense of safety (e.g., *How do you tune into other’s loss of safety?; Why does the sense of safety concept speak to you?* Or more specific questions prompted by previous findings like: *How does sense of safety impact connection to the body?*; or *‘How do you sense safety?*). Finally, they were asked to reflect and dream before discussing ways that the sense of safety concept could transform their approach to people in distress using metaphor, story, picture, or sensations with a prompt question: *What would it feel and look like in everyday clinical work once the sense of safety concept was in place? What needs to be different to make building sense of safety part of your everyday practice?*

### Data collection and protocols

Phase One: Between April and October 2017, five 90-min focus groups were facilitated by an experienced group facilitator with qualitative research training and experienced with managing patients and the different disciplines represented- the first author (family doctor JML). An experienced social science research assistant helped to facilitate group discussion. Focus groups ranged in size from three to 10 people and utilized written responses, post-it notes, and verbal discussion. Seven semi-structured interviews, lasting 90-min, were also conducted with two Indigenous academics, four rural general practitioners, and one rural mental health nurse. Each semi-structured interview and focus group was guided by an interview schedule. Phase One data focused on understanding the meaning of the phrase “sense of safety”—*the language*—and mapping the range of human experience that is relevant to understanding sense of safety—*the content.* It prioritized descriptions used by people who had a lived experience of loss of sense of safety in mental illness. It also started the process of understanding *dynamics* within a person that build safety—*the process.*

Towards the end of each planned topic guide questions (see above) for each focus group and individual interview, participants discussed and critiqued findings and emerging concepts from the previous iteration of the Sense of Safety concept as it developed. They were asked questions that explored concepts or specific words from previous participants, including ideas such as whether it is possible (or desirable) not to feel threat, how the concept of ownership of self was interpreted, and how well this concept fit with cultural safety.

An early summary of the findings from the focus groups was submitted as a draft document to the academic panel for review in July 2017. In March and April 2018, eight formal presentations of a later iteration were delivered for wider academic review and written feedback to international research groups in the USA (Chicago), Canada (Toronto), Norway (Trondheim and Tromso), and the UK (Liverpool and Hull). An early review of Phase One doctoral research results has been published (Lynch, 2021; Lynch, 2019). Raw data was reviewed again independently as part of Phase Two research.

Phase Two: Eleven 90–120-min online focus groups were facilitated by the first author (JML) and a psychologist (co-author KT) between November 2021 and July 2022 (ranging in size from one to 17 participants). Participants were invited to respond via a chat-box as well as participate in smaller zoom break-out room and whole group discussions. Focus groups were conducted online due to coronavirus restrictions as well as to facilitate the participation of interested clinicians who resided overseas. Each focus group was guided by a flexible interview schedule that was based around Appreciative Inquiry stages of define discover, dream, and design (Ludema and Fry, 2008). The semi-structured interview schedule was updated for each subsequent focus group as part of the iterative process. Phase Two re-explored the meaning and experience of sense of safety—*the language* and the *content* and then focused on refining understanding of the dynamics—*the process*—and how practitioners could facilitate or build sense of safety—*the practitioner response.* It included and valued the voice of a wide range of practitioners including a subset with self-identified experience of mental illness (mhc 1–3e).

### Data analysis

Focus groups and interviews were audio-recorded and transcribed (in two groups in Phase One, the patients were not comfortable being recorded and so we relied on written documentation and some researcher’s note taking to capture the content of those session). Other written documents (e.g., post-it notes, researcher notes, and chat box notes) were collated into the transcripts. Phase One analysis was undertaken by the first author (JML) with selected sections (approximately 20%) of the data independently coded by co-authors (MvD, CD, and PM) to compare coherence and reliability, and to discuss disagreements and reach consensus. Co-author KT also independently reviewed 100% of this Phase One data as another layer of analysis and identified two aspects that should be expanded on—the concept that safety over time is very important (with participants noticing that reflecting on safety or threat could make them feel younger) and the concept that freedom included freedom to ask for needs to be met. Phase Two analysis was undertaken by the first author with independent coding by a psychologist (co-author KT). In both phases the process of data analysis occurred in parallel to data collection influencing the iterative process. Data was collected beyond saturation in case new disciplines in later focus groups added further insights—saturation was determined by codes repeating with no new codes emerging.

In both phases, researchers followed the thematic analysis process outlined by Braun and Clarke (2006), first becoming familiar with the data, then conducting line-by-line coding to identify an initial set of codes. Transcript data was coded in small units, using gerunds (active verbs), memo writing, and axial coding to develop thematic conceptualizations. Codes were kept precise, simple, and grounded in the language of the data. Codes were *descriptive* (e.g., *what* was appraised to sense safety?) and *process-oriented* (e.g., *how* is safety sensed?; Saldaña, 2015). In the final analysis, the data was read and re-read from three perspectives: *what does a person experience when they feel safe?*; *what helps a person to feel safe?*; and *what can practitioners do to facilitate a sense of safety?* Participant voices were privileged in analysis; therefore, codes were repeatedly discussed with co-authors and eliminated if there was any evidence of theoretical abstraction away from the voice of the participants.

Second cycle coding was then applied to develop theoretical or pattern codes (Saldaña, 2015) and represent the research account (Crabtree and Miller, 2023) using the “Inclusive Logic” of transdisciplinarity in cycles of inductive, deductive, abductive, and intuitive reasoning (Lynch et al., 2020a; McGregor, 2004). In line with transdisciplinary understanding of knowledge as emergent, this search for patterns to discern what is most integral and coherent remained provisional and open to change throughout the research. Repeated spirals of consultation with co-authors, advisory panel, and wider academic critique across both phases exposed the analysis to the scrutiny of peers.

### Research quality and rigor

This research is based on transdisciplinary generalist approaches to knowledge that are built on broad inclusive scope of: participants and content; collaborative relational and participatory approaches to co-creation of knowledge (including presence of a second researcher at focus groups); an emergent attitude to provisional knowledge among the research team; the first author maintaining a reflective and reflexive attitude (including self-reflexivity on personal responses to the data in a written journal); and the frequent refocusing on pragmatic real world applicability of the knowledge as it formed.(Lynch et al., 2020a) Frequent conversations reflecting and debating on alternate interpretations of the data after independent analysis of sections of the data with co-authors, presentation of iterations of the findings to later participants, and formal critique by an international multidisciplinary academic advisory panel in Phase One all added to the reflexive and participatory rigor, transparency, generalizability and significance of this research.

## Results

Fundamentally, Phase Two and Phase One analysis were aligned. Data will therefore be presented as a building of understanding over time, with Phase Two data integrated into summaries of earlier iterations of Phase One data (Lynch, 2019; Lynch, 2021) if needed.

**Figure 1:**
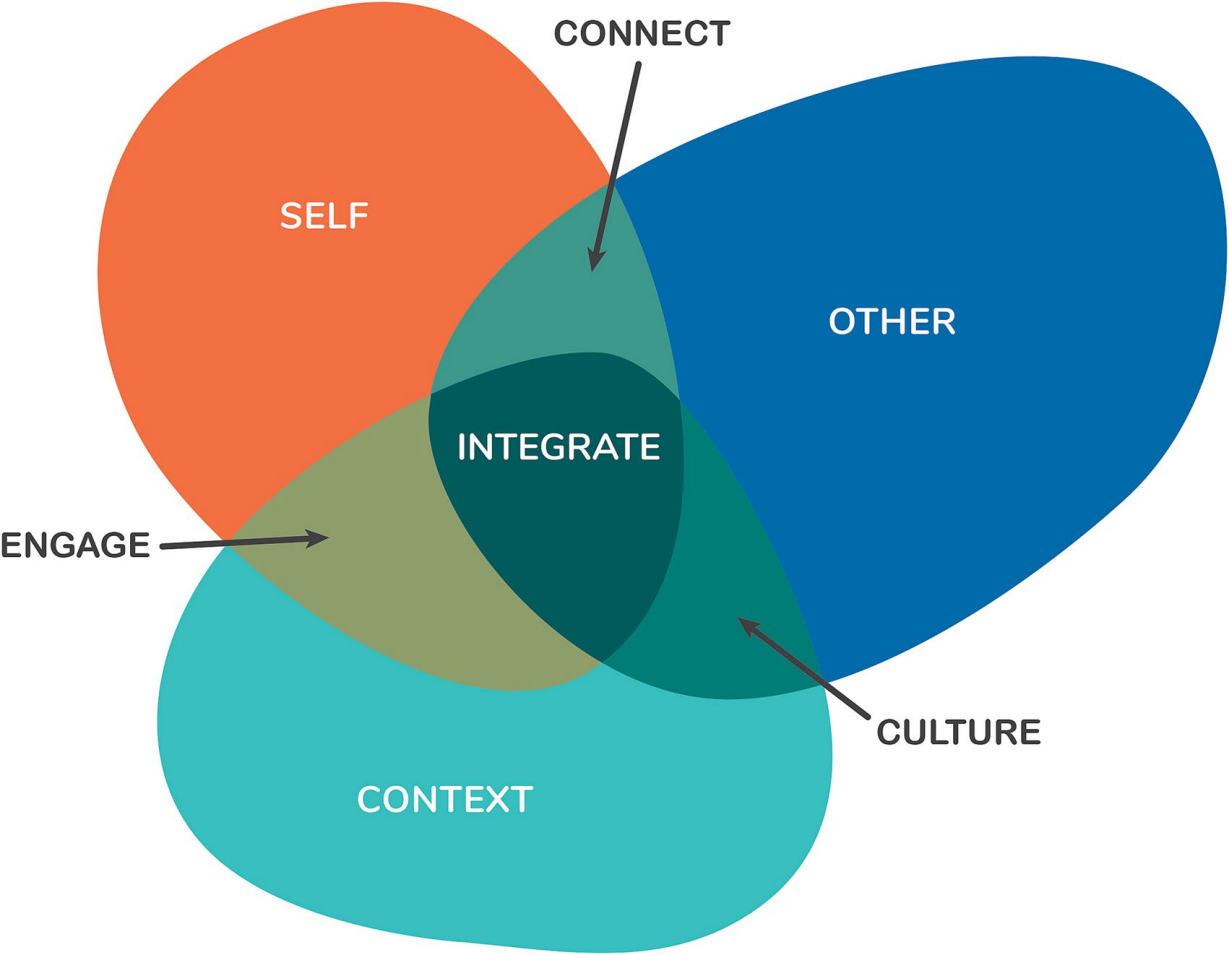
Defining the shared whole person language of sense of safety. Graphic representation of the responses to ‘what does the phrase ‘sense of safety’ mean to you?’ (note from Lynch (2021) A whole person approach to wellbeing: Building Sense of Safety. Routledge: UK).

**Figure 2:**
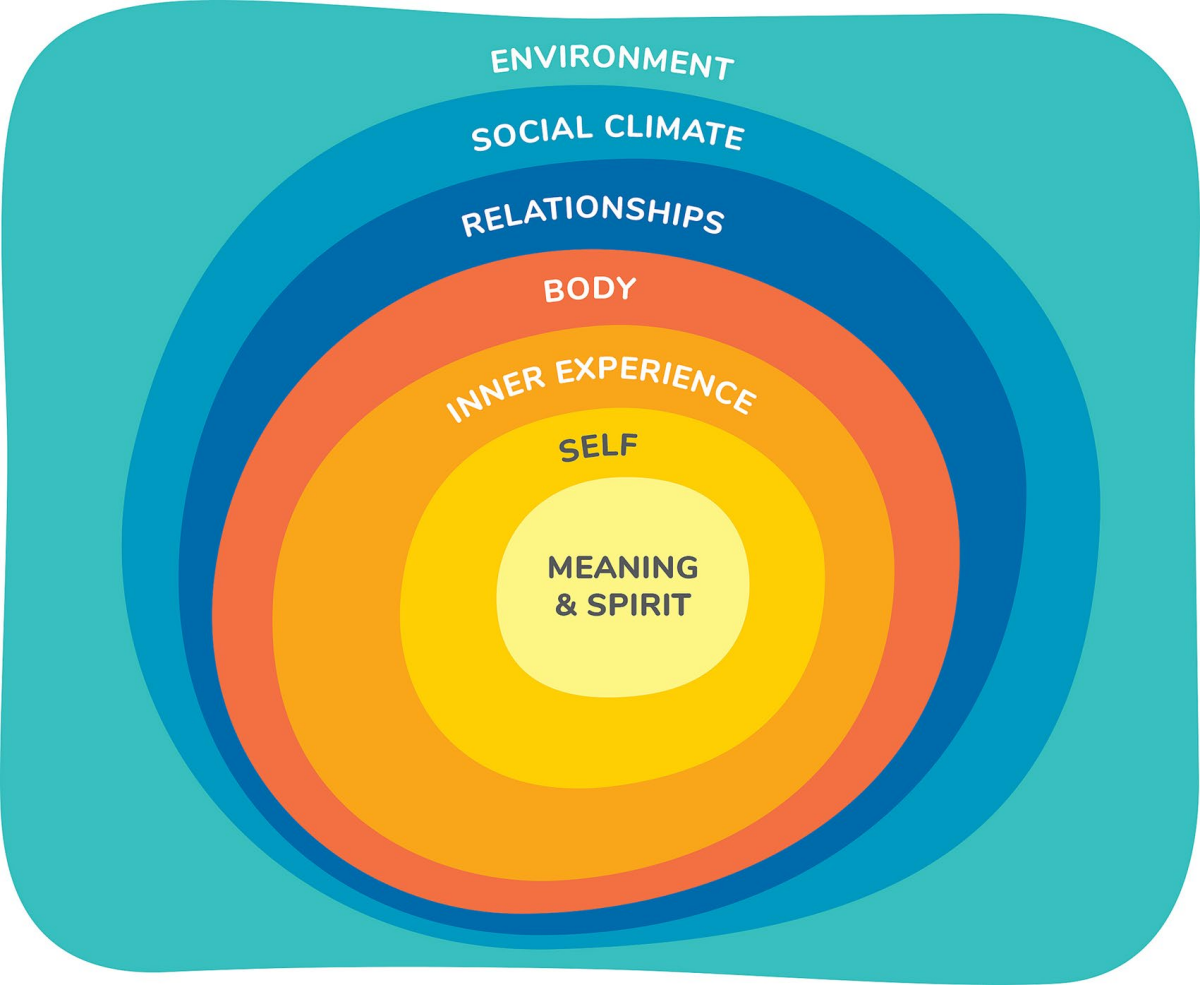
Whole Person Domains that define whole person scope of care. (Reproduced with permission from Lynch (2021) A whole person approach to wellbeing: Building Sense of Safety. Routledge: UK).

**Figure 3:**
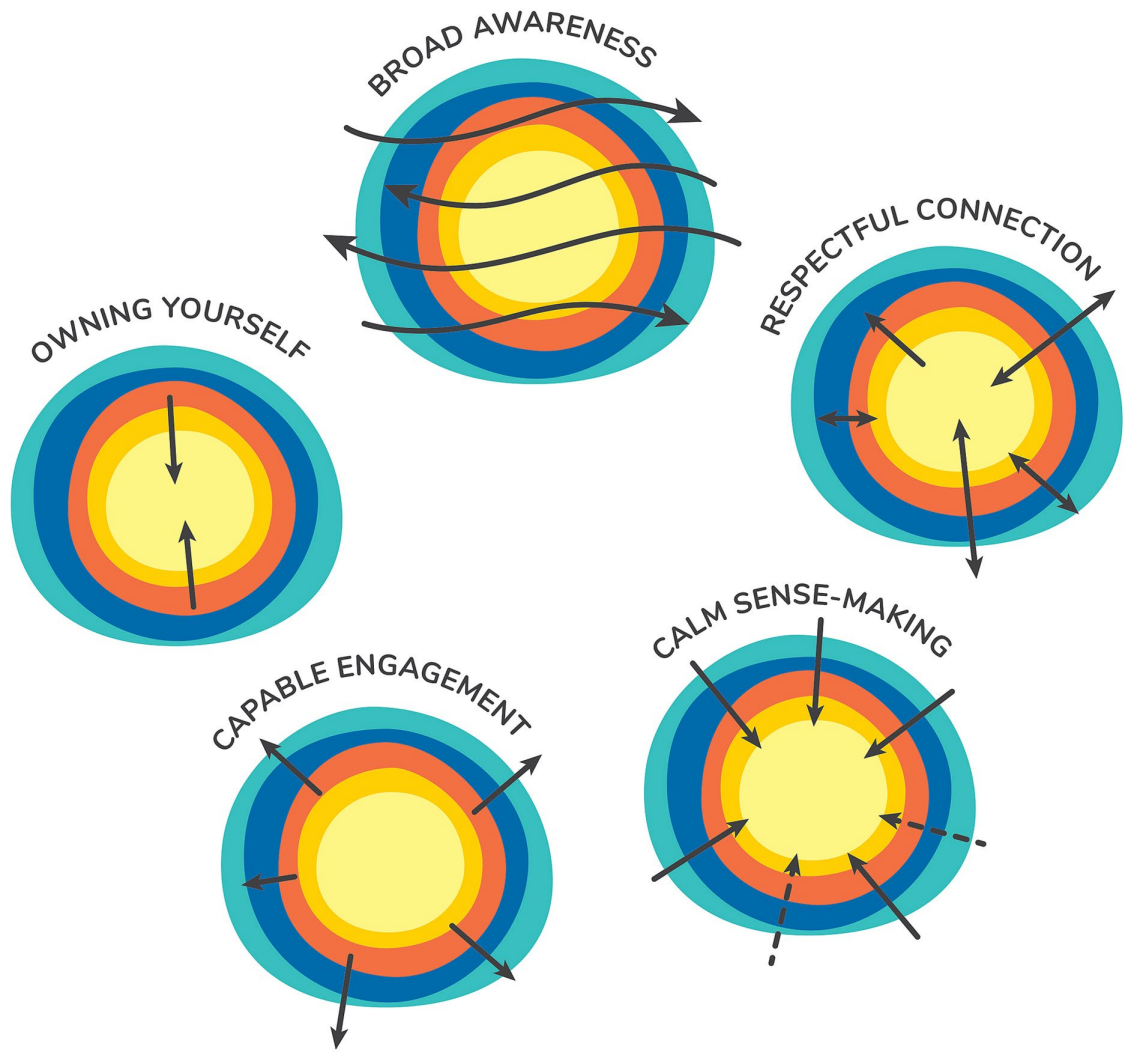
Naming healing goals. Representation of the Sense of Safety Dynamics as they occur across the Whole Person Domains. (Reproduced with permission from Lynch (2021) A whole person approach to wellbeing: Building Sense of Safety. Routledge: UK).

This results section describes four aspects of the development of the Sense of Safety Theoretical Framework. Firstly, it explores participant descriptions of the meaning of the phrase “sense of safety”—*the language* and describes the intuitive integrative whole person experience implicit in this ordinary English phrase. This includes Table 2 outlining the different layers of awareness and Figure 1 representing the overview of content and processes that were elicited just by asking participants what they though the words meant. Secondly, the results integrated any references to content in discussion of the meaning of sense of safety and explored responses to further questions in the focus groups and interviews that map the range of human experience that impacts sense of safety—*the whole person scope.* These results are made visual in Figure 2. Thirdly, the results explore the analysis of verbs and processes identified in the data that reveal dynamics that build sense of safety—*the healing goals.* These are represented in Figure 3 and outlined in more depth in Table 3. The multidisciplinary academic and participant critique of each iteration of the Sense of Safety Theoretical Framework is also included in this results section and positioned before later analysis. The fourth aspect of the Sense of Safety Theoretical Framework focuses on any identified, personal and cross-disciplinary practitioner skills that Phase Two participants described facilitating sense of safety—*the trauma-informed practitioner skills and attitudes.* These results are presented in Table 4. Each of these layers of analysis contributed to the shared language, broad scope, healing goals and trauma skills and attitudes that became the Sense of Safety Theoretical Framework.

**Table 2:** Themes and subthemes emerging from stakeholder written responses to the question “What does the phrase ‘sense of safety’ mean to you?”

Theme 1: awareness of how safety is sensed: Depth of Noticing	
Concurrent awareness of Self, Other and Context	*“Feeling secure within myself, my community and the wider world” (mhc)* *“Feeling safe*—*for my culture, spirit, identity” (ia)* *“feeling (emotionally and bodily) safe in this particular place, this particular time, with this particular person” (gp)* *“Feeling in control of own life, feeling of having supportive people around you, not feeling threatened*—*now or as a feeling that comes with you from your past” (mhc)* *“Freedom from fear, security, freedom from threats and intimidation; good mental health and physical health; people who love me and whom I love.” (le)* *“Not feeling any threat regarding your body, mind and spirit; feeling safe in all aspects of life, being respected for your mind, body, spirit” (le)* *“A feeling in yourself that you can be yourself and request for your needs to be met without being at risk of physical/psychological or cultural harm” (gp)*
Awareness of time - Past Present Future	*“not feeling threatened*—*now, or as a feeling that comes with you from your past” (mhc)* *“not at immediate risk” (mhc)* “not feeling threatened now…” (mhc) *“real sense of calm and peace and being present” *“freedom to be/function at my best level in the now and be able to look positively to the future” (mhc)* *“where negative impacts are identified, they can be addressed without ongoing harm, or fear of harm” (mhc)*
Awareness of context	*“Somewhere*—*a place a feeling that people can be themselves. A place/feeling free of judgement” (gp)* *“Individual feels comfortable in their environment” (mhc)* *“feel safe in this space” (gp)*
Awareness of culture	*“Secure in my culture, identity, environment and femininity without persecution” (ia)*
Awareness of relationships (including those not currently present)	*“people who love me and whom I love” (le)* *“feeling secure within myself, my community and the wider world” (mhc)* *“feeling of having supportive people around you” (mhc)*
Awareness of bodily experience	*“a feeling of wellbeing and calm and belonging without fear/anxiety” (mhc)* *“Being safe*—*and feeling that in all parts of my being” (ia)* *“being physically and emotionally comfortable” (mhc)* *“body signature” (gp6d)* *“But if you are constantly switching off, you are de-connecting everything in your body of who you are, you know, down to your core.”*—*(dfv2b)* *Be in your body –* *“…not too much sensory overwhelm” (gp4c)* *“…all this shame and fear and everything that stuck in their body. So I meet a lot of people who really do not feel a sense of safety.”*—*(o/g1f)* *“…there’s also a more blunt way that people connect to their body when they are stress/distressed with using high intensity exercise to control stress levels.”*—*(P8i)* *“By doing this cutting behaviour, she experienced some control again. So there was a sense of regaining that connection with your body and feeling that connection through that experience…. It was a staying alive thing”*—*(OT2i)*
Awareness of inner experiences and perceptions	*“Being safe*—*and feeling that in all parts of my being” (ia)* *“feeling secure within myself” (mhc)* *“a state of feeling calm, secure” (mhc)* *“not at immediate risk of physical or psychological harm” (mhc)* *“comfortable, feeling well and secure, the absence of threat” (mhc)* *“content within ourselves” (dfv2b)*
Awareness of self, identity, spirit and voice	*“feeling safe*—*for my culture, spirit, identity” (ia)* *“feeling safe in all aspects of life, being respected for your mind, body, spirit” (le)* *“it is very important for a person to feel safe within themselves” (gp)* *“people who do not like themselves do not feel safe” (gp)* *“secure in my culture, identity, environment and femininity” (ia)* *“sense of spirituality and connectedness in that sense of something bigger than me that can hold me… help you to feel safe… hold distress” (gp3d)* *“We talk about values more than we do now asking people what’s the value to them in their life, so spiritual but broader because for those who do not use the word spiritual, and that’s part of our job as clinicians sort of saying, what’s important to you?” (gp6c)* *“And I think I was reflecting on the, you know, my comment around this sense of safety being something that is you know, this transcendent quality, as maybe being quite a specific part of safety, like it’s a specific part of a sense of, of self around safety. And that that is something that is less negotiated with the external environment. Is something that’s more solid, internal, that people have a sense of safety around who they are, regardless of what external circumstance that they are in.” (mhc3e)* *“This is really hard because you are like there’s these very real existential threat. Now like I’m thinking 20 years’ time we know life as we know it may not be, you know, some of the Pacific Islands will be underwater…” (gp8c)* *“And we would say that spirituality is important for mental health as well. So I think there’s an element there of that a sense of something that’s bigger than you that can hold you and help you to feel safe.” (gp2d)* *“I think basically, your sense of meaning in life is actually really important in terms of been safe, even if you have terrible things happen to you.” (gp1c)*
Awareness of agency and direction	*“having a say in what is happening” (le)* *“I have the resources needed to deal with the demands of my environment” (mhc)* *“Sense of safety… an anchoring with a bit of anticipation of exploring new things… and a vibrant sense of being accepted and not criticised and able to go outside the lines.” (P3i)*
Integrated overall experience of safety (a kind of sense making)	*“Being safe*—*and feeling that in all parts of my being” (ia)* *“Feeling safe*—*for my culture, spirit, identity” (ia)* *“not feeling any threat regarding your body, your mind, spirit” (le)* *“entirely present and content" (gpP8c)*
Theme 2: awareness of what is appraised to sense safety: Broad and Concurrent Awareness	
Appraisal of self	
Awareness of threat to self in this context and relationships	*“sense that I am physically and psychologically safe”(mhc)* *“my life is not threatened” (mhc)* *“I will not be harmed” (mhc)* *“it is OK to be where I am” (gp)* *“a feeling of security, confidentiality, acceptance, absence of threat” (gp)* *“not at immediate physical or psychological risk of harm” (mhc)* *“that I will not be harmed” (mhc)* *“Keep it simple. Goal, keep it simple. Yeah. If you are not safe, you are not able to do or to hear or to, to take part in anything.” (gp4f)*
Inner sense of capacity to be and own themselves	*“An ownership of myself and my experiences, rather than being someone who is a bundle of symptoms and diagnoses” (le)* *“Feeling safe to be myself” (le)* *“A feeling in yourself that you can be yourself” (gp)* *“I can just be” (mhc)*
Appraisal of self in relation to context	
Confidence to engage with life and threat	*“it means an individual feels comfortable in their environment and in turn within themselves to step outside their comfort zone and try something new” (mhc)* *“feel free to try something risky” (le)* *“being able to function in a calm manner” (mhc)* *“I can meet expectations” (mhc)* *“I have the resources needed to deal with the demands of my environment” (mhc)* *“I guess one way of seeing is that is that when you feel threatened, when there’s a lack of, of security and the situation is meaningless and threatening. If you are unable to accept it and reorient yourself find meaning in a different situation than you wanted to be. And that triggers this demand for medications or investigations and so on. Even if at rational level you use, you should, you could have been able to appreciate that this is not going to help you I mean, those are smart people. Everyone is smart, really, but they are not in a situation and where they can appreciate, and I guess I’m seeing now that that comes down to lack of safety.” (gp8f)* *“looking after myself…I am working, I eat regularly, I maintain good health” (gp)* *“I have a goal is a protective factor, hopeful and will not give up” (gp)* *“feeling in control of my life” (mhc)* *“able to relax and thereby think more clearly, be more open to opportunity and able to be conscious*—*much healthier for being free in new situations” (mhc)*
Appraisal of self in relationship to others	
Inner sense of belonging and trust in the presence of others	*“being listened to and heard, with the sense/feeling that they are accepted and that they matter” (mhc)* *“sense of belonging” (le)* *“To feel relaxed in someone’s presence. To be comfortable in someone’s presence, to not have fear or anxiety. To feel trust in the others you are with” (gp)* *“But why oh, no one yet said anything about loneliness. Yeah, the most one of the most frightening things to be.”*—*(gp5f)*
Sense of freedom to express themselves	*“feeling safe to be myself” (le)* *“I can be myself and expose my gifts as well as vulnerabilities” (mhc)* *“where emotions of all kinds are okay and can be freely expressed” (gp)* *“feel accepted enough to express their vulnerabilities” (gp)* *“freedom to express myself” (mhc)* *“supported in order for me to have the freedom to be/function at my best level in the now and be able to look positively to the future” (mhc)* *“having a say” (le)* *“able to talk to someone openly” (gp)*
Appraisal of others	
Perceptions of the other person’s inner attitudes towards them	*“Being respected for your mind, body, spirit (le)”* *“Feel held and feel more whole” (gp)* *“Feeling acknowledged” (gp)* *“be accepted, nurtured, protected and encouraged” (mhc)* *“that I will be accepted and supported non-judgementally” (mhc)* *“feeling that what ever you do/say is treated with respect and confidence” (mhc)*
Awareness of other people’s supportive presence now and in the past	*“to have others to reach to when I’m overwhelmed” (mhc)* *“boundaries clear” (gp)* *“linked with well connected people who are “on my side,” right with me” (mhc).* “*I have a family who is helping me. I have good friends” (gp)* *“people who love me and whom I love” (le)* *“Feeling of having supportive people around you” (mhc)* *“loved by my family and friends” (mhc)*
Perceptions of other person’s character	*“trustworthiness” (le)*
Observations of the other person’s behaviour	*“having people who will listen without judgement or prior assumptions and form a connection around my experiences” (le)* *“someone who will listen, nurturing, caring, insightful, security, someone who’s on your side, someone who will journey with you” (le)* *“being with someone who will listen without judgement” (le)* *“feeling like I’m not being compromised or made vulnerable” (mhc)* *“taken seriously, acting in your (patient’s) best interests, listened to and feeling acknowledged, boundaries clear” (gp)*
Overall appraisal of risk in the other person	*“no danger/threat in the environment and persons” (gp)* *“a place/feeling free of judgement” (gp)* *“a feeling of security, confidentiality, acceptance, absence of threat” (gp)*
Appraisal of Context	
Global assessment of threat in environment	*“no danger/threat in the environment and person” (gp)* “*feeling safe in all aspects of life”* *“absence of danger/harm etc.” (le*) *“feel comfortable in their environment” (mhc)* *“I moved back from living overseas… there was a sense of familiarity, there was a cosiness… you know my senses had that familiarity in terms of the blue sky and the objects that were around and the energy with the people around me in that little community…. I felt this visceral release of okay*—*I do not have to hold it together anymore. I’m back where I know I can feel very nurtured and accepted and be myself and not have to work so hard to get through some of the basic things… it really rippled through my whole body and I wasn’t aware how much I’d been holding that sort of exterior bracing kind of posture.” (P9i)*
Place, space, situation feels safe	*“being given time and opportunity to know why a treatment is prescribed” (le)* *“away from where I feel unsafe” (gp)* *“I think safety is interesting when we talk about old people. Also interesting when we talk about old people because they might have the big lives and they thoughts, but the moment when they feel unsafe, they will give everything away for feeling a little bit more safe. –They will give up living home they will even move into a tiny room with a stranger if they know that when the night comes someone will have a look. I think even when we when we lose, lose things when we get older… it’s the safety that that is the last thing that we yeah, that’s the interesting aspects. I think. I think that’s interesting.” (gp4f)*
Environment provides adequate resources (including physiological needs, opportunity, time)	*“A place where the prerequisites for needs fulfilment is available” (le)*
Appraisal of Others in their Context	
Sense that culture is not threatened	*“Feeling safe for my culture, spirit, identity” (ia)* *“I had to kind of draw inwards to, to rely on my own cultural connections. So I know who I am I know where I’m from and know where I’m going. And regardless of the uncomfortableness around me, it enabled me to stand my ground.”*—*(Ru1k)*

Denotations in this document for each type of experience are: lived experience (le), Indigenous academic (Ia) family doctor (gp), obstetrician/gynaecologist (o/g), teacher (Te), physiotherapist (P), occupational therapists (OT), domestic violence support social workers (dfv), mental health clinicians (mhc), rural team (Ru), allied health primary health researchers (PC). Academic advisory panel members are denoted by an A ahead of the rest of the descriptor. Each focus group in Phase Two is denoted using the letters a-k.

**Table 3:** The dynamics of sense of safety: themes and subthemes that name healing goals of care.

Dynamic theme	Sub-themes	Example responses
Broad Awareness	Broad Scope of Awareness	*“Feeling secure within myself, my community, and the wider world” (mhc)* *“Real sense of calm and peace and being present” (gp8c)*
	Active Concurrent Awareness	*“Being safe*—*and feeling that in all aspects of my being” (Ia)* *“Sense of safety” does not mean safe or not anxious in any given moment. Sense is the general environment, but danger may erupt within an environment of safety. (gp7a)*
Calm Sense-Making	Being Aware and Clear-Headed	*“Bodily sense of calmness, being able to think clearly and creatively” (mhc)*
	Notice Broadly	*“I know how to recognize that I feel threatened” (gp)* *Things could make sense in that moment. Like I could feel a connection to something like to nature, something bigger than myself.—(mhc1e)*
	Know Intuitively	*“I will go down into this, I will start to compute inside myself and I’m not even aware of it” (Ia)* *“what is your gut telling you?”—(dfv2b)*
	Organise and Make Sense	*“Filter it through the maps in their mind” (gp)* *“Having the capacity and ability to be able to assess your place in the world” (dfv2b)*
Respectful Connection	Available and Trustworthy	*“Presence, attachment*—*not too close or too far from the other” (mhc)*
	Tuned Into	*“Somebody that gets you and that you can test out your perceptions with” (mhc)*
	With you/On your side	*“Loving supportive attachments in all social spheres” (le)* *“peacefulness and calmness and feeling secure enough to be able to speak without having to give a second thought”—(gp6c)*
Capable Engagement	Freedom to Move/Grow /Learn	*“Given the opportunity to learn and ‘fail’ and be encouraged to keep learning” (mhc)* *“Sense of safety means having free choice about how I spend my time and with whom, with freedom to come and go as I need to, and having people both within that space and outside that space with whom I feel comfortable that I am understood and will be treated with respect”*—*(gp4d)* *“Movements very, or lack of movement, when people are injured, is very connected to confidence. And that’s not just physical confidence in terms of making your way physically in the world, but feeling confident about all aspects of where you fit in the world. And I’m thinking particularly of older people, when they feel a bit weaker, or they their balances a bit off, and they do not move as much and feeling like quite restricted in their opportunities to, to get out and about. And so I think movement and confidence with movement are deeply connected to how people feel in themselves in their own sense of safety.” (P8i)* *“…we do not get narrative writing our of children experiencing distress. We can get some formula writing out of them. But the space to imagine and generate ideas and dream… that will not happen” (Te1h)*
	Having a Say	*“You feel safe enough to just state your perceptions about things” (mhc)* *“being free to express one’s self without fear of judgement or censorship and being able to have an authentic exchange of ideas”*—*(gp6d)*
	Positive Direction	*“Meaningful work or creativity” (gp)* *“knowing that things will turn out right… even if it feels rough right now”*—*(gp1f)*
Owning Yourself	With Yourself	*“With ourselves” (le)* *“I would say the owning yourself domain, like for teenagers, especially that ability to feel really confident in yourself can vary widely. And you can usually see the kids who are very much confident in their own bodies and confident in their selves and their personalities. And you can usually tell the students who maybe aren’t confident yet just this sort of thing like that, you know, it’s puberty everyone goes through a stage I think when they are maybe not feeling that inner self clearly.” (Te4h)*
	Asking for my Needs Capacity Acknowledged	*“getting even better at being in tune with my needs and asking for them and then feeling that they might be at least listened to even if not addressed. … at least we can negotiate it in place and without rejection even if someone cannot meet my needs. Least I can acknowledge them…” (gp6c)* *Love, needs met, permission to be who I am, opportunity to grow (gp1a)* *“To be comfortable and able to fully express my needs and self*.”—*(gp1c)* *“Agency to address threat… agency to make your work safer” (gp)*
	Comfortable	*“Able to relax, reduce monitoring my environment” (mhc)* *“That gave me the sense of safety and yo8u use that word, lose yourself in that you are not watching yourself anymore… going back to those elements of being a freedom and authenticity like not having to feel like I need to be who other people expect or want me to be… a place where I can truly be me.” (mhc1e)*

**Table 4:** Practitioner skillsets and attitudes that build sense of safety: summary.

Theme 1: Valuing the whole picture (to facilitate Broad Awareness)	
Value a generalist gazee
Attentive to multiple broad complex aspects of life over time
See the system
Aware of systemic obstacles to and providers of safety in power structures and clinical environments (socio-political, organisational, clinical)
Tune in to both bodies
Intuitive discerning and dynamic ‘gauging’ – trusting your gut for accurate perception – sensing changes in both people
Include paradox
Concurrent awareness of both discomfort and safety, congruence and incongruence
Theme 2: Holding Story Safely (to facilitate Calm Sense Making)	
Invite the story
Model and normalise journeying together towards knowing and accepting reality
Hold and contain
Organising multiple realities at once
Soothe and co-regulate
Set pace, flow, and direction to bring comfort to discomfort, laugh together, normalise
Join a dance
A generous and tenacious dance of presence and validation
Integrate wisely
Seeing coherent patterns that facilitate healing across a spectrum
Theme 3: Being With You (to facilitate Respectful Connection)	
Be comfortable with not knowing
A position of humility that accepts not knowing and imperfection without second guessing or assuming
Be present:
Listen with your heart (dadirri) in a tuned in relational dance
Have their back
Trustworthy acceptance and commitment to stay and support
Repair ruptures
Be vulnerable enough to reconnect and manage power
Take care
Take care with small moments, words, no words, and time
Theme 4: Learning Together (to facilitate Capable Engagement)	
Hold space for collaboration
Increase capacity through working together
Rebuild boundaries together
Build capacity to articulate and negotiate – emboldened to stand their ground
Envision a future
Hold hope and meaningful connection to something bigger
Seed safety
Plant seeds to grow and reach out to others
Believe in them
Believe in their internal value and capacity to rebuild themselves
Theme 5: Protecting Dignity (to facilitate Owning Yourself)	
Welcome and invite
Include all parts of the person
See and value inherent personal dignity
Help them to be comfortable in their own skin
Remind of capacity
Build capacity to tune into their own intuition and make their own choices
Draw inwards and centre myself too
Care for self as person, sensor, container, reflector, integrator, healer

### What does the phrase “sense of safety” mean to you? Analyzing the whole person language

Analysis of written responses to the question *What does the phrase ‘sense of safety’ mean to you?* explored the utility and generalizabiliity of the language “sense of safety.” Participant responses in both phases revealed a concurrent awareness of self, other, and context. For example, a mental health clinician described “sense of safety” as “*feeling secure within myself, my community, and the wider world*,” while a family doctor described it as “*feeling (emotionally and bodily) safe in this particular place, this particular time, with this particular person*.” Responses also revealed an integrative concurrent awareness of content and processes or dynamics between self and others (connection), self and context (engagement), and other and context (culture) as outlined in Figure 1.

The “connect” dynamic is revealed in the following response: “*to feel relaxed in someone’s presence. To be comfortable in someone’s presence, to not have fear or anxiety. To feel trust in the others you are with*” (gp). The “engage” dynamic is exemplified in these words: “*it means an individual feels comfortable in their environment and in turn within themselves to step outside their comfort zone and try something new*” (mhc). Finally, the “culture” aspect is evident in the phrase: “*feeling safe in my culture, spirit, identity”* (Indigenous academic—Ia).

Written responses also revealed an integrative process—where the whole organism is attuned to threat in any aspect of the whole: “*being safe*—*and feeling that in all aspects of my being*” (Ia); and “*not feeling threat regarding your body, your mind, your spirit*” (le). responses from both clinicians and people with a lived experience reveal a dynamic process of broad concurrent awareness (named “Broad Awareness” and discussed below) that seems to be an integral aspect of sensing safety.

All participant responses revealed a depth and breadth of noticing, including an awareness of context, culture, relationships, bodily experience, inner experiences and perceptions, self, identity, spirit, and voice, agency, and direction, as well as past, present, and future threat. This range of content was noted and integrated into the second aspect of analysis below. This noticing seemed to integrate sensing and sense-making into an overall sense of safety that was aware of dynamics within themselves, and dynamics between themselves, others, and their context as revealed in Table 2. Sense of Safety is a *whole person language*.

### What causes threat? Analyzing the content to define *whole person scope*

All participant conversations in response to open questions and discussions were analyzed for any clues as to the breadth of human experience (the content) that contributes to threat or sense of safety. An analysis of these responses in Phase One resulted in the naming of seven themes that were confirmed in Phase Two. These themes were named “Whole Person Domains” to draw attention to the way they intersect and interconnect as part of the whole person experience of threat and safety such that no single domain can stand alone. These intersecting parts of the whole are depicted graphically in Figure 2 and are then each considered in more detail below. They include *Environment, Social Climate, Relationships, Body, Inner Experience, Sense of Self, and Spirit/Meaning*. Phase Two analysis confirmed these domains as mapping the landscape of where threat and safety are experienced across the person. This analysis defined the scope of whole person car as part of the Sense of Safety Theoretical Framework.

“Environment” was considered to encompass a “*safe place to sleep*” (mhc) and “*it will either feel ok or not ok in that environment*” (Ia). This domain included subthemes of potential threats (or resources) from: the *physical environment* (including climate and Indigenous connection to country), *lack of time and information*, and *lack of equity*. Participants described the influence of politics, finances, uncertainty, and freedom and stability in the workplace, school, and/or other social and physical environments. Phase Two participants emphasized this with conversations about systemic sociopolitical, organizational, and healthcare environments that threaten safety.

In terms of “Social Climate,” participants clearly articulated the importance of the social atmosphere at home, where living and learning happen, and in the wider culture and community (including social media). This drew attention to living situations, migration, change, remoteness, noise, addictions, criminalization, racism, pessimism, finances, job availability, language, rituals, and intergenerational trauma. Phase Two analysis strengthened awareness of sense of safety in the workplace.

The role of “Relationships” is captured in the phrases “*others to reach out to when overwhelmed*” (mhc), “*accepted, nurtured, encouraged*” (mhc), “*feel held and whole*” (gp), and “*intimacy and equality in relationships*” (mhc). They confirm the ways that personal relationships contribute to feeling safe. Participants noticed *who* is in the person’s life, *what* interactions they have (including loss of relationships), and *how* those relationships are conducted (including tone of voice, trust, attunement, and reliability). Participants mentioned family, sexual relationships, friends, carers, neighbors, children, parents, extended family, coaches, bosses, and therapeutic relationships.

The general themes of threat from relationships included *disconnection* (loneliness, exclusion, loss, abandonment, disengagement, and being shamed and disregarded), *invasion* (confrontation, disrespect, violence, intimidation, bullying, and other forms of abuse), and *confusion* (confusing relationships, injustice, betrayal, and being misheard or misunderstood). Sub-themes of experiences of safety in relationships were described as: being heard and understood; belonging; trust; a sense of meaningful support; being able to be “messy” and have big emotions and ask for needs to be met without fear of rejection; and being treated with dignity.

The “Body” domain included sub-themes of noticing the *physical body*; *movement and behaviour*; *awareness and sensation*; and *capacity for regulation*. These ranged from appearance, body language, mobility, and facial expressions, to temperature, heart rate, allergens, disease, and physical distress. Sleep, diet, substance use, medications, exercise, hormones, emotion, mobility, and aging were also mentioned. Participants mentioned the influence of genetics, personality, temperament, and sensitivity to stimuli (including touch and needles). Phase Two participants mentioned the “*ability to listen and feel into their body*,” and mentioned a kind of “*gauge of how vulnerable I can be*” *(dfv4b)* and capacity to “*recalibrate*” *(dfv2g)* in the body. They also mentioned “*bringing them back into their body*” *(dfv1g)*, or “*regaining that connection with your body*” *(OT2i)*, intuitive “*checking in with ourselves*” *(gp13a), a* “*dance of soothing*” *(gp2d),* and “*body signals*” *(OT5i)*.

Key experiences participants described that were relevant to loss of “sense of safety” included arousal, intoxication, shame, hunger, pain, physical impact of a lifetime of extreme stress and violence, foreboding, hypervigilance, flooding emotions, too much responsibility, and anxiety. The inner experience of illness or cancer as loss of control was described as “*threat from within*” (mhc). Others said “*the body holds everything*” (Ia) and described the importance of a capacity to “s*ense bodily calm*” (mhc). Phase Two participants also mentioned the use of “*high intensity exercise to control stress levels*” *(P8i)* and described “*shame and fear and everything stuck in their body*” *(o/g1f).*

The “Inner Experience” domain included descriptions of the subjective inner world of the person. Overall subthemes were of *peace* (or shame, uncertainty, and hopelessness), *connection* (or feeling disconnected, invaded, avoidant, numb, exposed, or vulnerable), and *inner organization and reflection* (or confusion, loss of control, intense emotion, compulsions, powerlessness, pain, and fear). This domain included thoughts, memories, mood states, interpretations, perceptions, sensations, intuition, self-talk, and attention. Phase Two participants described being “*content within ourselves*” *(dfv2b)* and a “*fluidity of being able to kind of go in and out*” *(le),* “*ebb and flow*” *(Te1h),* or “*circle back*” *(dfv1g)* to assess internally.

In the “Sense of Self” domain, the inner communication and attitudes towards the self that participants described are captured in the words of a family doctor who said: “*it is not safe if you do not like yourself*.” Overall, sub-themes of safety were inner attitudes of *dignity*, *trust*, and *unity*. Inner attitudes of respect, integrity, trust of self, connection with self, acceptance, worthiness, stability, confidence, not having to second guess yourself, and feeling loved were mentioned as aspects of the self that contribute to a “sense of safety.”

The “Spirit/Meaning” domain included participant descriptions of both a sense of personal meaning and fulfilment and any spiritual, religious, or existential beliefs or concerns. Overall sub-themes included *hope and purpose*, *sense-making*, and *connected experience*. Participants described a sense of knowing “*who and why you are*” (gp). They named a capacity to explore or “*create your own meaning about your life story*” (gp), as well as a sense of transcendence in soul or spirit, culture, and country to “*sense something that’s bigger than you that can hold you and help you to feel safe*” *(gp2d).* Loss of hope, loss of existential security, shame, loss of faith, fear about the future, and disrespect about your own beliefs were threats mentioned by participants.

### How do you sense that you are safe? Analyzing the *processes* to name *healing goals* that build and protect sense of safety

All participant conversations in response to open questions and discussions were analyzed for any clues as to the active verbs used to describe a sense of safety or threat (the process). Alongside the dynamic of “Broad Awareness” (with sub themes of *broad scope* and *concurrent awareness*) already identified from the initial question about the meaning of the phrase “sense of safety,” four other dynamics were identified that build, protect, and reveal a sense of safety. “Calm Sense-Making” captured themes of *being aware and clear-headed*, *noticing broadly and knowing intuitively*, and *organizing and making sense*. The response “*computing inside myself*” (Ia) captures this theme. “Respectful Connection” included sub-themes of awareness of the quality of connection with others as *available and trustworthy; tuned in*; and *with you/on your side*. “Capable Engagement” included sub-themes of *freedom to move, grow, and learn*; *being able to have a say*; and *being able to move in a positive direction*. This is captured in the response: “*I have the resources needed to deal with the demands of my environment*” (mhc). The final dynamic noticed in the participant responses is “Owning Yourself.” This dynamic was captured in the phrase “*sense of safety is owning yourself and your experience*” (le) and reflects sub-themes of: a *sense of being with yourself*; *having your capacity acknowledged*; and *feeling physically comfortable*. Phase Two analysis added: *asking for my needs*. If these dynamics are dysregulated or absent, participants described a loss of “sense of safety.” These dynamic themes are summarized in Figure 3 and Table 3.

### What do participants think of the emerging understanding of sense of safety? A critique

When participants and the academic panel (respondents) were asked to critique the emerging understanding of sense of safety, sub-themes emerged of it being *potentially useful*: “*I think you are onto something, and it is something that needs to be shared. It is our job but we have not been taught how to do it*” (gp) and “*sorely needed*” (gp), as evidenced by one mental health clinician stating “*safety, this is it, this is the treatment, this is your job, it is not just a complication*”(mhc). One family doctor said it was “*cultural safety for everyone*” (gp).

Respondents also spoke about the concept being *out of the comfort zone*: “*what you are talking about is a paradigm shift*” (GP) and “*this is a complete reframing of what we think about people and their context*” (GP). They therefore expressed *translation concerns*: “*I think it’s going to make a huge difference… it’s a fabulous idea… but it’s just how do you embed it?*” (Ia). One family doctor was concerned about the potential that the focus on safety might lead to more risk-averse approaches to health saying “*life is not about safety*—*challenges require that you feel unsafe a lot of the time…”* (gp), while an Indigenous academic said “*I think it captures the idea of safety really well. It sounds familiar, if that makes sense, like a lot of it is probably common sense… it’s quite accurate*” (Ia). A mental health clinician on the academic panel described it as “*intriguing and coherent*” and another wrote:

*I think Sense of Safety is a lovely phrase*—*it is common English*—*it works*—*everyone thinks they know what it means*—*and probably everyone’s idea of what it means is not too different from what everyone else’s idea of what it means*—*so it is useful*. (mhc)

This analysis identified the potential usefulness of the phrase “sense of safety” and the concepts of threat and sense of safety. It also identified the underlying uncomfortable paradigm shift of this approach and voiced concerns about the real-world potential for it to be translated into clinical practice or training. This directly influenced the questions about integrating sense of safety into everyday practice asked in Phase Two.

### What do practitioners do to facilitate a sense of safety? Analyzing the practitioner *skills and attitudes*

**Figure 4:**
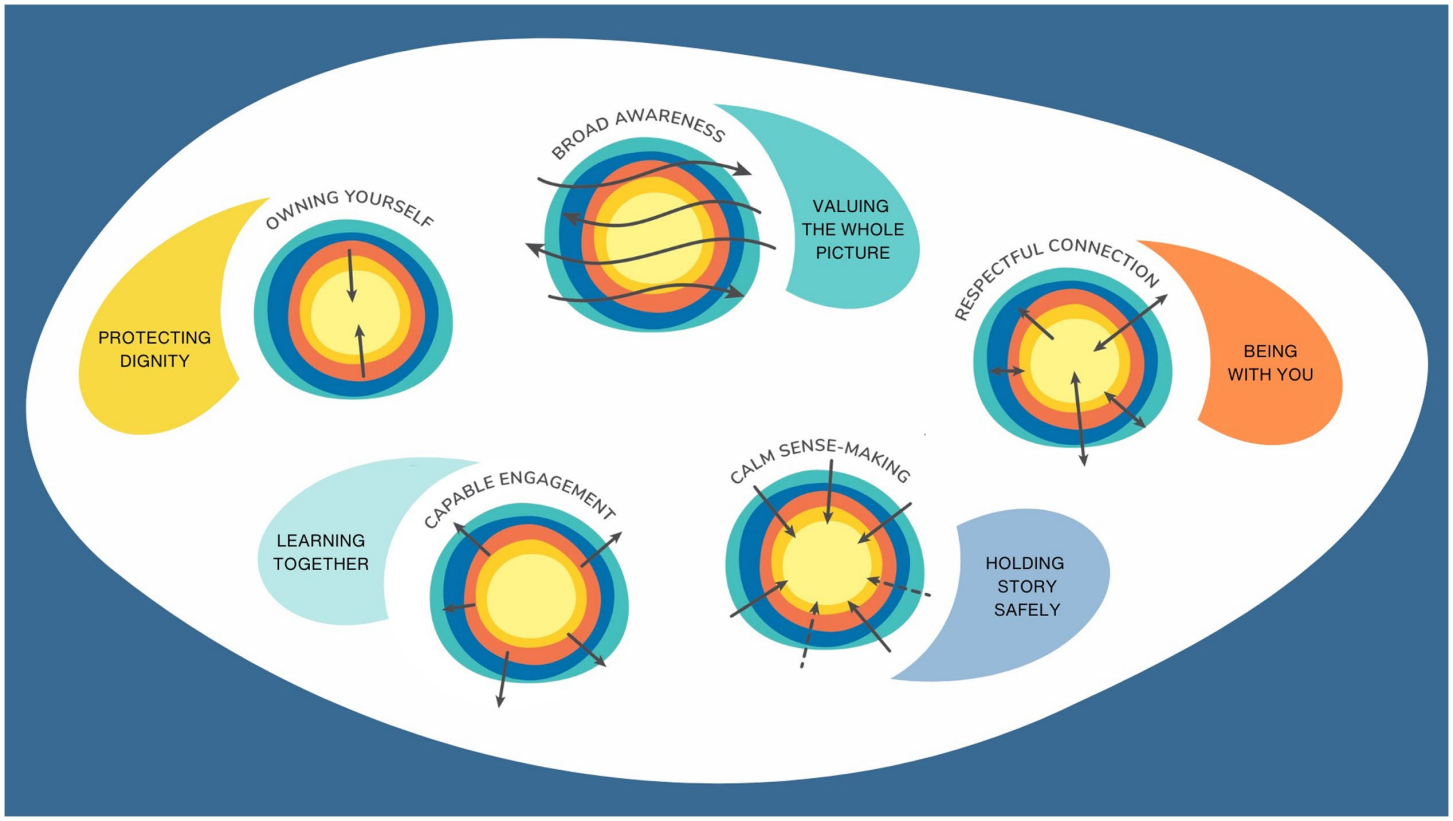
Practitioner skills and attitudes that build sense of safety dynamics.

As outlined in summary Table 4 (and Supplementary material), and made graphic in Figure 4, clear themes emerged from the conversations with multidisciplinary practitioners about the way sense of safety could influence practice. These processes naturally aligned with or facilitated the Sense of Safety Dynamics in Figure 3.

### Each theme describing a skill and attitude also had subthemes

Key practitioner skills and attitudes that build and protect sense of safety were identified as *Valuing the Whole Picture, Holding Story Safely, Being With You, Learning Together, and Protecting Dignity*. Each of these skills and attitudes facilitated a Sense of Safety Dynamic as noted in Table 4, clarified in the Supplementary Table and depicted in Figure 4.

Participants described a way of being that facilitated “Broad Awareness”: *Valuing the whole picture*, with subthemes of (a) *value a generalist gaze*, (b) *see the system*, (c) *tune in to both bodies*, and (d) *include paradox*.

Participants described ways of listening and making sense of story that facilitated “Calm Sense-Making” in both clinician and patient. This skill was named *Holding Story Safety* and had subthemes of (a) *invite the story*, (b) *hold and contain*, (c) *soothe and co-regulate*, (d) *join a dance*, and (e) *integrate wisely*.

Another skill identified was *Being With You*, with subthemes of (a) *be comfortable with not knowing*, (b) *be present*, (c) *have their back*, (d) *repair ruptures*, and (e) *take care*.

Skills that built sense of capacity were named Learning Together and had subthemes of (a) *hold space for collaboration*, (b) *rebuild boundaries together*, (c) *envision a future*, (d) *see safety*, and (e) *believe in them*.

Participants descriptions of skills that build capacity for ‘Owning Yourself’ were named Protecting Dignity, with subthemes of (a) *welcome and invite*, (b) *see and protect the person’s dignity,* (c) *remind of capacity*, and (d) *draw inwards and center myself too*. These themes and subthemes, drawn from natural discussions, point to practical ways that paying attention to sense of safety could influence the processes of everyday practice.

## Discussion

This study explored the concept of sense of safety from differing and widening perspectives across both phases of the research. Initially asking what the words “sense of safety” meant to participants—*the language.* Then seeking to understand the range of experience of sense of safety—*the whole person scope,* and dynamics that build sense of safety—*the healing goals*—from participant descriptions of both sense of safety and threat. Then asking practitioners what they already did or dreamed of doing to facilitate a sense of safety—*the practitioner skills and attitudes.* As well as participant feedback on previous iterations of analysis, at two key points in Phase One, an academic panel critiqued the findings giving insights into the potential usefulness of this concept in real world practice. These multiple approaches to the concept from different practitioner and lived experience perspectives have led to a rich understanding that has become the Sense of Safety Theoretical Framework, including an understanding of the *language* of “sense of safety”; the mapping of broad *whole person scope* relevant to appraising sense of safety; the dynamic *healing goals* that build sense of safety; and the *practitioner skills* and attitudes that facilitate sense of safety.

Sensing that we are safe is a fundamental multidimensional prerequisite for health. Analysis of participant descriptions of what the phrase “sense of safety” meant to them revealed a complex integrative awareness of self, other, and context. Sense of safety was described by participants as a process of appraising capacity to “engage” at the same time as quality of “connection” to other people in that context. This aligns with research that highlights the role of appraisal of coping and perception of social support as part of sensed safety (Matthieu and Ivanoff, 2006; Teicher et al., 2016; Timperio et al., 2015). Sensing we are safe is a moment-by-moment in-built embodied response to internal and external stressors and resources. This means that the ordinary phrase “sense of safety” is a naturally sensitive and integrative whole person experience and strength-based language shared by practitioners and the people they care for. Appraisal of Sense of Safety has the potential to become a shared language, a collaborative goal, and a broad map for whole person care of distress.

Asking participants about threat and how safety is sensed allowed us to map the range of influences on sense of safety relevant to health, defining the whole person scope. This awareness of “what” caused threat or loss/lack of sense of safety led to clear domains of the whole person relevant to appraising sense of safety. These Whole Person Domains are named: *Environment, Social Climate, Relationships, Body, Inner Experience, Sense of Self, and Spirit/Meaning making* (See Figure 2). They represent a full range of social determinants of health such as injustice, housing, finances, political instability, food insecurity. They also represent relational safety in community influenced by hopelessness, addiction and poverty, and personal relational safety influenced by experiences of being tuned in to and protected. This aligns with the WHO definition of safety and as a “perception of being sheltered from danger” (Maurice et al., 1997, p. 181). The domains also integrate awareness of physical safety, and internal experiences of inner unity, organization, self-respect and meaning making aligning with research into sense of self and the importance of personhood in health (Dowrick et al., 2016). Despite being very different aspects of the person, the Whole Person Domains revealed within the concept of “sense of safety” offer a unifying transdisciplinary way to assess people’s wellbeing. Because these domains attend to threat—they are a trauma-informed way to define whole person care. Multidisciplinary practitioners found these concepts to be useful and sorely needed, signaling the potential for this approach to be a practical and comprehensive systems review that facilitates cross-disciplinary communication and contributes to public policy priorities by defining scope of care.

Asking participants about threat and how safety is sensed allowed us to notice patterns or processes (verbs) that could contribute to healing and health, by naming healing goals of care. Participants described active dynamics that built, protected and revealed sense of safety. These Sense of Safety Dynamics were relevant across the whole person, including self, other, and context. They included *Broad Awareness, Calm Sense-Making, Respectful Connection, Capable Engagement,* and *Owning Yourself* (see Figure 3). Pattern recognition is an antidote to the fragmentation of single-disease frameworks and guidelines (Muth et al., 2014) that currently overwhelm clinicians as they try to understand the impact of psychosocial issues, and interactions between diseases (Dowrick, 2004). Improved recognition of patterned responses to loss or lack of sense of safety could improve early diagnosis and intervention in complex disease progression. Conversely, inadequate recognition of these complex patterns leads to late diagnosis, fragmented diagnostic and treatment processes, costly incoherent care, and health system miscommunication (Sturmberg et al., 2021). These healing goals align with the new Research Domains Criteria (RDoC) neurological frameworks of ‘Arousal and regulatory systems, Sensorimotor systems, Perception and understanding of self/others; Attention; Perception; Cognitive systems; Systems for Social process and Positive valence systems.(Cuthbert and Insel, 2013; Lynch, 2021) These whole person dynamics also clearly link sensed safety to engagement and action—a link between comfort and courage—sensing we are safe enables normal development, learning, growth, healing and reconnecting with life and community. The patterns identified in this study as Sense of Safety Dynamics offer new therapeutic directions for quality care.

When we asked practitioners what they already did or dreamed of doing to offer care that facilitated sense of safety their responses corresponded to theses dynamics. Practitioner Skills and Attitudes emerged: *Valuing the Whole Picture, Holding Story Safely, Being With You, Learning Together,* and *Protecting Dignity* (See Figure 4). These active Practitioner Skills and Attitudes clarify first principles of helpful trauma-informed approaches to distress in healthcare, education and the social services sector, and link them with defined healing goals of care. Named in this way they are much more than humane approaches to care, they are fundamental to the function of healthcare, education, and social services practice—they change physiology and enable fundamental shifts in health risk when they increase a person’s sensed safety. They define and guide best practice, training, and policy design that is trauma-informed and healing-oriented.

The Sense of Safety Theoretical Framework as an approach to healthcare, education, and social services research, and public policy is in its infancy. Future research could focus on developing measures of sense of safety in different target populations, cultures, and contexts, and developing new therapeutic techniques built around attending to the Whole Person Domains, facilitating the Sense of Safety Dynamics, and teaching the Practitioner Skills and Attitudes. Understanding the breadth of the whole person impacted, and knowing what helps to facilitate Sense of Safety in both practitioner and patient could transform practice, facilitate transdisciplinary communication, refine research approaches, set new treatment goals, and define often implicit or assumed clinical skills required to build sense of safety. It could also transform safety in healthcare and education by prioritizing subjective safety that soothes and comforts in addition to current objective attempts to ensure few things go wrong (Safety I) or many things go right (Safety II; Hollnagel et al., 2015).

The Sense of Safety Theoretical Framework is a potentially unifying approach—relevant across the whole person, grounded in wide transdisciplinary research, integrating many fields of research, and translating that into a practical way of seeing that captures both objective and subjective influences on the person and their health. It is a multilayered theory that facilitates attention to the whole person. It integrates awareness of the impact of social attunement and social determinants of health as well as internal embodied experience. It is a framework that acknowledges the inherent appraisal systems that assess capacity to cope, presence of threat, coherence of meaning, and perception of social support. Sensing safety impacts our health from communal relationships down to a deep cellular level. The language and concept of “sense of safety” is embodied, relational, and meaningful to us all—the person in distress, their clinicians, and the wider community. It is a strength-based and healing-oriented approach designed to increase community and practitioner capacity to orient towards healing and build sense of safety across the whole person. It highly values both comfort and courage – both healing and growing. It offers a new way to tune in to the person’s lived experience and physiological responses to the multiple layers of threat and safety in a community. Sensing safety can integrate past experiences of harm or security and remain aware of future hope and capacity. It is trauma-informed, and oriented towards healing across the whole person and community.

At its root, the word “safety” comes from a Proto-European base word: *solwos* which means whole (Nilsen et al., 2004). The word “healing” comes from the Old English word “haelan” which means “to make whole” (Harper, 2020). This study translates a wide body of literature and cross disciplinary consultation into a practical framework for practice. It includes an analysis of the language, the relevant range of enquiry into the whole person within their context (Whole Person Domains), the processes that build sense of safety (Sense of Safety Dynamics), and the ways that practitioners can facilitate those processes (Practitioner Skills). We therefore propose that ordinary English phrase “sense of safety” operationalized as the Sense of Safety Theoretical Framework may offer a way to map and unify our understanding of health across the disciplines. This framework is a trauma-informed and healing-oriented approach to whole person care that could help to make our community whole.

## Data Availability

The raw data supporting the conclusions of this article will be made available by the authors, without undue reservation.
