# Supplementary material for: The sense of safety theoretical framework: a trauma-informed and healing-oriented approach for whole person care
Source: Front Psychol. 2025 Jan 14;15:1441493. doi: 10.3389/fpsyg.2024.1441493 (PMC11772489; doi:10.3389/fpsyg.2024.1441493)
Supplement: Supplementary file 1 [file Supplementary_Table_1.docx]

Supplementary Table: Themes and subthemes practitioners describe that facilitate sense of safety. Each skill and attitude facilitates a corresponding Sense of Safety Dynamic. Subthemes name aspects of each skill.

**Theme 1: Valuing the whole picture (to facilitate Broad Awareness)**

| **Value a generalist gaze:** attentive to multiple broad complex aspects of life over time | *‘guided by her story… build the whole picture up … put it all together… then we can really see clearly …really unpack and understand the whole picture’ - dfv1g*  *‘it’s a very broad and rich thing’ – mhc2e*  *‘This is a big and complex concept”- gp8c*  *‘I think that one of my main roles being a GP, for people over time, is to contribute to safety. Sometimes I think it's the only thing I do for some of my patients is to be the safety.” -gp7f*  *‘It is a chronology of time, and its also a perception thing, but it grows on you…it evolves as you grow older... there's a timeline thing that's really important, and so the story starts very early on.’ – P6i*  *‘To make part of my practice - integrate it more into a holistic view of the person, where would not just talk about social safety but the broader picture (at the moment I think this is really siloed into different professionals for different bits of the person’s life).’ - dfv5a*  *‘So, we think of the relevant part of life as being our medical work, but for most of our patients, most of their cares and troubles and choice are outside the office.  You know, they want to go bowling and go to church and see the kids and other stuff and it's easy to to not pay enough attention to those parts of their lives.’ – gp11a*  *‘…if we could see the small signs we never talked about and he's dead… and he still he still helps me a lot to think about him and to to know that he wouldn't hurt if he was here. Gives me a sense of safety still.’ – gp8f*  *‘And that was the that was the first time she she realised that something like that happened. So, so, it can be deep deep down there, right?’ – o/g1f* |
| --- | --- |
| **See the system:**  aware of systemic obstacles to and providers of safety in power structures and clinical environments (socio-political, organisational, clinical) | *‘I think practically having like a shared understanding of the world, what safety is, and then how to achieve that across sectors...being able to have that shared understanding and that respect, across services and sectors to be able to support people would be a dream." - dfv6a*  *‘I also wonder if the clinic /space you are working in actually plays a huge part. The practice culture needs to be about connecting with all staff/Drs and this vibe will then welcome the patients.’ - gp5d*  *‘and it starts with the team, you know, it starts with the team and feeling like you have the support of your team, so that you can go out and, and give that care’ – GP13a*  *‘the frame provides the safety – the antithesis of chaos’ – gp6d*  *‘time-based medicine… doctors worried about being sued…litigation and finances’ – gp5d*  *‘...it'd be great in 20 years time, if we valued relationships more than achievements. If bullies had consequences, rather than rewarded with with promotions, or were just sort of, we avoided conflict and let them get away with it. If we do things to minimize the trauma that affects the safety, but that's a dream.’ - gp6c*  *‘huddles, you know, like asking a question like, what's on your mind today, as we approach our work today, with a reminder, it's about our patients, but it's about ourselves too- clear the decks and then 'let's go'.’ - gp7a* |
| **Tune in to both bodies:**  Intuitive embodied discerning and dynamic ‘gauging’ – trusting your gut for accurate perception- sending change in both people. | ‘*My sense of safety is the level I gauge in how vulnerable I can be in any given situation.’ – dfv4b*  *‘Being very attentive to change’ - P6i*  *‘…being really reflective and aware.’ - dfv1g*  *‘Bring them back into their body.’ -dfv1g*  *‘we're sort of exploring whether they noticed within themselves’ – P9i*  *‘ vigilant for the ones who have been numbing, and who have that reduced ability to listen and to feel into their body.’ – P3i*  *‘ I might be completely unaware that someone is, you know, acquiescing to me a a therapist but not really coping at all with what I ask them to do…if people can gauge their own level of distress… I just need to be aware of their body language a lot of the time.’ – OT2i*  *‘responsiveness…. ebb and flow…Matched affect…physically near – proximity’ -Te1h*  *‘it's a response in yourself to like, it's, I call it my gut feeling. It's my intuition, my gut feeling. But obviously, I'm responding to something right in front of me , but I just almost feel like, I can feel it. So I it's so hard to describe.’ - gp2d*  *‘In stepping out of the comfort zone or stretching beyond what I feel like normally… I think I do an internal check in around how I'm going on a embodied sense. If I notice that I'm feeling really heightened. I'm a bit keyed up and nervous, like, oh, maybe it's not safe enough. Maybe my body's telling me it's not safe enough. – mhc1e*  *‘ if their response is like, very strong, then it kind of gives an indication that they already are living on the edge, they already don't have any buffer remaining.’  – Te2j*  *‘Sometimes that process of approach to provide comfort and closeness is threatening in itself, especially if the child feels like the parent has triggered the distress in the first place.’ – OT5i*  *‘…like one naming what those body signals are, and to accurately identifying like, oh, that body signal means this emotional response… sometimes it comes to like, I couldn't tell the difference between butterflies in my tummy versus physically feeling sick… or having parents saying, like, you're not actually sick, stop pretending to be sick. Like, when they are having a physiological response. They're just not able to name exactly what it is like, what are the hugely invalidating experience for them.’ – OT5i*  *‘quite a simple concept but then reflecting on how its really transient and …ephemeral … it just depends on so many factors….gp8c* |
| **Include paradox:**  Concurrent awareness of both discomfort and safety, congruence and incongruence | *‘feel deep sorrow and sadness while still feeling safe’ - le*  *‘I think it is possible to be distressed and safe’ – gp4c*  *‘Still feeling deeply angry or whatever the emotion is whether we deem it positive or negative but still feeling incredibly safe ‘. - mhc3e*  *‘different to the sense of safety that comes with doing somethng that makes you feel better…taking drugs, drinking alcohol, not eating… ways people do things to feel better - different to a true sense of safety" – gp3d*  *‘To be comfortable and be able to fully express my needs and self.’ – gp1c*  *‘am I not assessing this correctly… looking back at your tools, at the patterns and behaviour… there’s something more here’ -dfv1g* |

**Theme 2: Holding Story Safely (to facilitate Calm Sense-Making)**

| **Invite the story :** model and normalise walking together towards knowing and accepting reality | ‘*And all I did was sit with her and listen to her. And when she ran out of things to tell me. I told her stories, you know.’ – gp 16a*  *‘...begin the encounter by simply asking 'what's happened to you'... what's happened to you, and then just wait for the story...their story takes precedence over whatever clinical story you're going to put together.’ – gp17a*  *‘That sense that we're in this together for the long run, I think, I think for me, their safety in that. And I think for the patient, there might be that as well. And in special circumstances, when there's a scary diagnosis or uncertainty of the future, that notion that I'll walk this path with you wherever it goes. Now, that that could be a patient who's facing a cancer diagnosis or something that's frightening to them. The notion that I'm your doctor, and I'm with you, and wherever this goes, I'll be able to take care of you that exudes a certain kind of comfort and confidence.’ - gp11a*  *‘I'll walk this path with you wherever it goes...I'm with you, and wherever this goes, I'll be able to take care of you that exudes a certain kind of comfort and confidence. -gp11a*  *‘I’m going to walk alongside, I’m not going to try and fix you, or you know mold or change it, I’m just there being open-handed.’ -P3i*  *‘We haven't called out continuity. But I think that's very important, not just the relationship stuff that I appreciate, know about. But there's at least two components of that. One is the sense that sharing with a patient that I'm with you here, we don't need to do this all today. We can work on this over time.’ – gp11a*  *‘He sat with me for two years, and he never gave up on me.’ – gp16a*  *‘But again, what I like in the context that I really appreciate now is that actually, the relationship with my patients is just with something simple with a vaccine with a deep thing, but the deep things, all the complex stuff still comes out in the moment of this encounter, if you take the moment to be patient…. I'm just thinking of all the COVID vaccines we've had to try and talk people into, you know, but they have been relationship building moments where someone's brought up something else. And I've realised they're really stressed about something and then I booked them in, come back and see me in a week but in the moment, I still had to spend 5-10 minutes being present and creating the safety there to bring them back.’ - gp2d* |
| --- | --- |
| **Hold and contain:**  organise multiple realities at once | *'what you see on the surface level can have lots of contributing factors and if we're trying to address certain factors and there is no change, it probably means we are addressing the wrong thing' - dfv5b*  *‘And being open to what emerges in the moment, trying to stay in that moment, also, during the encounter, and bring our own life experiences, not just our academic experiences, kind of as a whole person, as a clincian, healer to that space.’ - gp6a*  *‘So you'll get children to retell - and we talk about characters and then talk about the setting and what happened, and then what happened next, and what happened in order… but others the retell comes out jumbled - when we are listening we wonder did it get jumbled going in, or get jumbled inside, or get jumbled coming out? Was it jumbled the whole way? – Te1h*  *‘ is being able to have compassion to be able to have understanding and to be able to draw the link to, or this is why I feel frozen, I can't do anything. It's because you know, when, when I was small and didn't have the capacity and resources to be able to manage that... And if I can draw that link, then that can gives me a way of, I guess going back to your model away of meaning making your main way of making sense of my experience now... My experience is very different to when I was younger, and I can do something differently about it now’. – mhc1e* |
| **Soothe and**  **co-regulate:**  set pace, flow, and direction to bring comfort to discomfort, laugh together, normalise | *‘sense of clearing a space in me to notice carefully - slowing down physically and cognitively.’ – gp10c*  *‘Have the the ability to coexist with discomfort and bring comfort to it’  -gp12a*  *‘Be an unnaturally calm person who can’t be rattled… slow down, speak slower, speak more calmly…nothing you say will shock me or make me go away..’ -Te1h*  *‘safe enough to connect, safe enough to proceed’ -gp10c*  *“fluidity of being able to kind of go in and out” -le*  *‘It's really about slowing down. Everything like slowing down process, and doing …what you know, helps to create a sense of safety. If you don't, then the process is slower.’– dfv2g*  *‘dance of soothing - you create the safety in all sorts of ways’* ***–*** *gp2d*  *‘It's like, yes, they're safe now. They're trying to get rid of all the things that their bodies stored up that they couldn't express when in an unsafe environment, and you just happen to be the environment for that.’ – dfv5a*  *‘grounds me and helps me integrate the crappy stuff from my day’ – gp2a*  *‘..we don't need to do this all today. We can work on this over time.’ - gp11a*  *‘...patience will heal our patients…’- gp6a*  *‘My sense of safety is the level I gauge in how vulnerable I can be in any given situation.’ – dfv3b* |
| **Join a dance:**  a generous and tenacious dance of presence and validation: | *‘Offer co-regulation so that the dance of presence and validation and empathy by being there and holding space for our inner experience, so we are not portraying any distress. Sending that message that your distress, your inner distress isn’t distressing me. I can hold space for that… maintaining your own sense of stability and calmness to kind of co-regulate’ -OT5i*  *‘I’m here to walk alongside you, I’m not going to pull you forward, I’m not going to pull you back.’ -dfv1g*  *‘Talking about the patient doctor relationship, and the sense of safety, or the sense of trust that someone had mentioned also that’s what I am trying to do in my daily work…. It’s the feeling of being seen… I’m here for you…I think that much of our job is to give our patients a sense of safety.’ – o/g1f*  *‘I guess this has been a reminder to look for signs of a 'sense of safety' in students. It is too easy to get caught up in trying to deliver the curriculum, but if students are not feeling safe, they are not going to engage with the curriculum anyway so it is important to gain that sense of trust and safety first. Even if they haven't felt safe in other places/classes, I always want them to feel safe in my classroom and makes me more cognisant of trying to establish that sense of safety.’ – Te2j*  *‘As a GP I feel that I fight unsafe. It’s really a tough world. Its never knowing what is going to happen. I might do mistakes, and so on.’ – gp6f*  *‘...we almost need other people in order to heal, like in order to, to be able to to, I guess feel safe or to be able to work through things...’ mhc1e*  *‘I think that sometimes the most important thing we do... part of care is ... is to accept the discomfort and pain of the patients... to let them exhibit fear or anger or even anger with us... and you just have to sit there part of its witnessing, and part of it is saying I can tolerate this and and I think, I think we're serving the patients often when we feel more uncomfortable because they're unloading on us, and we see we get a better view into their pain of that experience and, and what's interfering with the interaction right now. So that's just part of the work sometimes it's hard work but but sometimes it's fulfilling because it's kind of a breakthrough. It certainly contributes long term to a relationship’" - gp11a* |
| **Integrate wisely:**  see coherent patterns that facilitate healing across a spectrum | *‘I also think they the client or the person in front of you, so much happened in that initial moment of meeting, that it's obviously in a circular pattern. As you sense them and sense you and something emerges out of that, and to try and capture it in words. I think you may I think I lose that after that actual moment.’ – gp1d*  *‘I feel like there's not like safe and unsafe, it's like a spectrum . And I had to see it like that a little bit easier, because I don't think there's very many times when I'm like, yes, I am 100% safe right now.’ - Te4j*  *‘circle back… assess a pattern of risk and pattern of behaviour’ -DFV1g*  *‘tension and precarity of safety… slipped through my fingers… tension and contradictions of the sense of safety’ - le* |

**Theme 3: Being with you (to facilitate Respectful Connection)**

| **Be comfortable with not knowing:**  a position of humility that accepts not knowing and imperfection without second guessing or assuming | *‘clarifying, all reflect on what’s going on and not just make assumptions’ -Ru4k*  *‘Thinking the more we assume that we know what is going to make somebody else safe, the less safe that person is actually going to be because we're imposing upon them.’ - gp8c*  *'what you see on the surface level can have lots of contributing factors and if we're trying to address certain factors and there is no change, it probably means we are addressing the wrong thing' - dfv5b*  *‘Yeah, it almost seems like there's there's like potential for a tipping point that it swings on a balance, that sense of safety is is always in the presence of precarity. That sense is is is there in relationship with a precarious situation of some sort, whether we call that threat or whatnot. But it's sometimes that that in swinging in that balance, it can tip over into a very unsafe experience in precarity’. - mhc3e* |
| --- | --- |
| **Be present:** listen with your heart (dadirri) in a tuned in relational dance | *‘Being really reflective and aware’ - dfv1g*  *‘space where I had somebody there who was prepared to listen’ – gp4d*  *‘I was thinking, as you're saying that you're trained you, you you’ve verbalised what I was struggling with because I, we've been talking about how, how we see things and how we feel things and what we sense in like the dance, but it's actually, it's actually more 3D than that, because we're actually we're actually creating safety, as well.’ - gp6d*  *‘There is an internal sense of ‘contentment, settledness or capacity to hold distress if present’. – gp3d*  *‘But why oh, no one yet said anything about loneliness. Yeah, the most one of the most frightening things to be.; I don't think anyone wants to be alone in their suffering. [GP5f] mentioned loneliness in the in the context of getting old but but there is this broader or more existential aspect to names loneliness. So being alone and be he lost and afraid in suffering is terrible’. – GP5f* |
| **Have their back:** trustworthy acceptance and commitment to stay and support | *‘someone having your back. And the relational, the relational sense of security’ -gp1a*  *‘we are a social tribe’ – ‘a huddle’… ‘I can have your back when you are nervous’ - gp1a*  *‘sense of not having to be alone with responsibility’ – gp8f*  *‘loneliness… one of the most frightening things to be’ and…. safety is: ‘having someone I can ask for help’ – gp5f*  *‘safest I’ve ever felt was the moment when a hand slipped into mine’ -mhc3e*  *‘And emotions that are not constraining motions that don't lead to a sense that I need to mask or need to behave in a certain way’ – mhc3e*  *‘Just sitting in thinking about this sense of safety and being a GP and concepts and being practical, and we are doers, we do things. And I think that one of my main roles being a GP, for people over time, is to contribute to safety. Sometimes I think it's the only thing I do for some of my patients is to be the safety. They know, they can contact me, they know I can't solve the problem, but I will be there. And I will be there over time… And I think the GP the most valuable, valuable part of being a GP for patients with with palliative needs, it's not being the expert on pain medication, and it's being the safety, the being the one that knows the patients that knows the family that knows the system that is available. So I struggle a little bit about the concept of sense of safety, but um,* ***I think I'm good at doing it****. And I think that a lot, a lot of my colleagues also we do it, but we don't have the words or the language. But when we talk to each other in my office in the afternoon, after all the patients have gone home, and we sit down and have a cup of tea and and talk to each other. These are the things that we talk about,* ***what did I do today for somebody you at least made some safety for?*** *You understand? Yeah. I just wanted to say that. – gp7f* |
| **Repair ruptures:**  be vulnerable enough to reconnect and manage power | *‘when we’ve passed a little sudden hiccup there’s a lot of laughter and sharing of humour, to sort of, its almost like an acknowledgement that we’re back in a safe space’ gp1c*  *‘fragile safety…’ ‘sense of safety is always in the presence of precarity’ -mhc2e*  *‘When I'm working with someone who is experiencing trauma and feeling really unsafe, I will ask for consent to touch at every point. So it is continual negotiation of that sense of safety with that person, rather than just the beginning.’ – P3i* |
| **Take care:**  with small moments, words, no words, and time | *‘But to be honest, if I'm really honest with myself, I'm just over words and language. I think it's such you lose so much in the process of dialogue. It invites so much interpretation that it really gets in the way of being and experiencing what might be moment to moment going on for either.’ – gp1d*  *‘taking the time to actually ask the deeper questions and the further questions’ (dfv2g)*  *‘I think we would sort of you know, if it was say that the feelings of being unsafe the bodily sensations that go with feeling unsafe are just so much more in your face. You know, there's so much more overwhelming and alarming. So that was the sense I think in which feeling safe is almost it's a much stiller kind of space.’ – GP10c* |

**Theme 4: Learning Together (to facilitate Capable Engagement)**

| **Hold space for collaboration:** increase capacity through working together | *‘creating a space to learn together’ - Ru1k*  *‘hold that space in that way which is about collaboration and connection and partnership, as opposed to coming in as some sort of expert’ - OT2i*  *‘routinely stop every few minutes and say ‘how are we going?’ - OT2i*  *‘people are partnering with you and supporting you and encouraging you’- Ru5k*  *‘agency about and in their life’ – dfv1g*  *‘Yeah, look, I'll just take what it makes me feel like quite excited and hopeful talking about broadening that sense of safety for people and where they can experience that. And I think it just makes me feel sort of really hopeful and excited that you're working on this. And, you know, for me, it's a sense of just collaboration. And that whole that thing comes to mind that you know, domestic violence, domestic and family violence is everyone's business. And I think you know, this kind of work is really making it everyone's business. And it's just increasing people's capacity, because you don't know what you don't know. And no matter who you are, or where you're working until you know better, then you can do better. And I think any kinds of increasing capacity to know how to connect with people to establish that sense of safety is incredibly powerful and valuable.’ -dfv2g*  *‘free of intrusion, free of coercion… freer to articulate… willing to have a different view..’- mhc 2e* |
| --- | --- |
| **Rebuild boundaries together:** build capacity to articulate and negotiate - embolden to stand their ground | *‘enabled to stand my ground’ - Ru1k*  *‘clearly articulate my boundaries … set that tone… be empathic and hold space… but know where that line stops’ – dfv1g*  *‘I can have boundaries and I can put protective things in place for myself’ - dfv1g*  *‘Sense of safety means having free choice about how I spend my time and with whom, with freedom to come and go as I need to, and having people both within that space and outside that space with whom I feel comfortable that I am understood and will be treated with respect.’ - GP4d*  *‘protective interrupting’ – dfv1g*  *‘But I often say to women, what is your gut telling you? And that's the boundary that you want to put in place, if you're saying that, that you're not comfortable with that, or that you don't want to be in that place with that person.’ – dfv2b*  *‘...ability to be aware of what you need in that moment is also really important part of that.’ dfv1b* |
| **Envision a future:**  hold hope and meaningful connection to something bigger | *‘give people a little bit of hope’ - dfv1g*  *‘productive struggles… push through it but I’m here for you’ - Ru5k*  *‘...what do you want that to look like? Who are you as a person, as a woman… So, a certain freedom has to kind of happen for someone to be able to even envisage a life or a future that they want.’ - dfv2b*  *‘I know, purpose and meaning for me is crucial. I think that that it's something that I'm always, always needing to find a sense of something, something bigger or something, some purpose behind everything. I think it's one of my more annoying traits that I have. Is, is always feeling like I need to search for, for purpose or make purpose out of things. And I think I was reflecting on the, you know, my comment around this sense of safety being something that is you know, this transcendent quality, as maybe being quite a specific part of safety, like it's a specific part of a sense of, of self around safety. And that that is something that is less negotiated with the external environment. Is something that's more solid, internal, that people have a sense of safety around who they are, regardless of what external circumstance that they're in’. – mhc1e*  *‘So he has a meaning in life. He has something which makes sense to him, keeps him from suicide, which is quite far from what I could really relate to him on a deep level, but it's really deep to him because he has lots of, nothing is safe in his life.’ – gp6f*  *‘Just when I think about safety in my own late life, its related to meaning’ – gp4f*  *‘I think basically, your sense of meaning in life is actually really important in terms of been safe, even if you have terrible things happen to you.’- gp1c*  *‘knowing that things will turn out right… even if it feels rough right now’ – gp1f*  *‘Freedom to be able to envisage a life or a future that they want’ – dfv2b* |
| **Seed safety:**  plant seeds that grow and reach out to others | *‘Sense of safety: Love, needs met, permission to who I am, opportunity to grow’ -gp1a*  *‘having permission to be myself’ – P3i*  *it looking at the strengths for someone and building on the strengths of what they already have to influence their sense of safety?’ - gp11c*  *‘I think you can land on growing environment that's safe, so you can be anxious about doing something new or extending skills but the person who sets the scene for that or facilitates it can do that in a way that makes it safe.’ – gp6c*  *‘We’re in a place to keep trying making mistakes, and we can keep going, and I can stretch you further.’ – Te2j*  *‘But I'm really clear in my mind now, which I wasn't when I was younger. That my role as a GP is a, is a consult that goes on for you. Yes, it's not just this moment in time, actually the seeds I plant now I've seen they come, they grow up five years, 10 years later, you've all had patients who said, you remember what you told me 10 years ago? Like, whoa, okay, you know, but again, I feel like the chaos of it I'm really comfortable with now. So I agree with all those issues, you know, running on time demanding people difficult stuff, I guess it's that feeling that the safety and the healing we're building is in the small moments of a viral UTI or COVID vaccination conversation...’ – gp2d*  *‘This has reminded me that the students who are isolated in the classroom are likely not to be feeling safe. Or if they have only one friend. This makes me think about spending curriculum time on attempting to make the students feel safe with each other (rather than just with me). Feeling safe with each other (plus with me) will, in theory, give them the confidence to foray out into the unknown and try to learn something difficult.’ – Te3h* |
| **Believe in them:** their internal value and capacity to rebuild themselves | *‘grown-up who believes in them, and absolutely is communicating that positivity and belief’ -Te1h*  *‘how are you going to fix this problem? What are you going to do? Some action coming out of it that’s actually an opportunity to believe that they can be part of the solution’ -Te1h*  *‘a kind of ordering yourself or putting together’ – dfv 2b*  *‘But the point is that he needed the safety of me believing in him, and recognising what he's done, and that he's holding it together.’ -gp2d*  *‘give people choice… transparent around consent process’ (dfv1g)*  *Phrases like 'I'm here if you need me' things that allow them to know that you're with them. And that you are there to provide closeness and care without necessarilty needing to be the one to initiate and giving back a little bit of control. - OT5i*  *‘ he needed the safety of me believing in him and recognising what he's done’ – gp2d*  *‘Sense of safety means having free choice about how I spend my time and with whom, with freedom to come and go as I need to, and having people both within that space and outside that space with whom I feel comfortable that I am understood and will be treated with respect.’ – gp4d*  *‘Confidence and control over what you were about to choose to do’ – dfv5b*  *‘Baseline of you’re safe and confident’ - dfv5b*  *‘Able to manage this risk’ – dfv4b*  *‘Comfort and confidence’ – gp 11a* |

**Theme 5: Protecting Dignity (to facilitate Owning Yourself)**

| **Welcome and invite:**  include all parts of the person | *‘feel they are truly seen and heard… seeing every part of them show up however they want to do that’ - dfv2g*  *‘build each other up… model failing, that we feel unsafe, we stuff up, show them its ok, still going ok … even celebrating – how did we fail this week – give each other a round of applause’ - Te4j*  *‘that healing brave space for allowing some vulnerability, and not just for our patients, but ourselves’ – gp6a*  *‘Listening to the story beyond labels’ – gp 3d*  *‘And so, letting people share their story and asking them, Tell me your story. I find that's valuable in terms of patients who are suffering with chronic pain, or in patients who have mental health disorders. Just allowing someone to name that instead of taking all these biases that we get from reading the electronic medical record and other people's opinions can really be powerful for patients.’ - gp6a*  *‘you’re safe now, and your finally no longer in fight or flight survival mode, and you can actually feel the emotions you have’ – dfv3b*  *Not having to second guess your response – gp6c*  *having people who completely get where you're at, and there's no need to be stoic, no need to be on your number one game the whole time – OT2i* |
| --- | --- |
| **See and value inherent personal dignity:**  help them be comfortable in their own skin | *‘Being free to express ones self without fear of judgement or censorship and to be able to have an authentic exchange of ideas.’ - gp6d*  *‘Being able to be yourself, without any kind of change or adaptation, without fear.’ –dfv5a*  *‘Yep and there is no need for you to change who you are in order to be loved, accepted, understood.’ - gp3d*  *‘being reassured of who you are, where you sit, and your sort of purpose and journey through life’ - Ru3k*  *‘I don't I don't feel like my credibility will be questioned when I speak you know, I can go you know, I'm I don't think that people will question my relationship with my partner.’ - mhc3e*  *‘Being with someone without trying to fix it or correct it - just being validated for who you are and what you are saying.’ – OT5i*  *‘...we accept them even when they make a mistake or don't know what to do, or when they have a different idea or direction than others do.’- gp8a*  *‘Safe to be vulnerable and grow as well’ – gp6c*  *‘Because I think, for me, when I feel absolute safest as probably when I can be most emotionally vulnerable. And those two may not always align with your natural tendency towards what does safety look like in a pretty picture. And might actually just be really, really messy because I actually feel safe to be messy.’ - dfv4a* |
| **Remind of capacity:**  to tune into their own intuition and make their own choices | *‘I need to coexist in this world of, as we said before, tensions moving quickly from safety to unsafety. But there are things I can do to build, build my sense of safety.’ – mhc2e*  *‘I think that the best moments that someone has had in my room is when we're not doing anything, and then we get a sense of how to find their own centre , and knowing how to access that and being that they go.’ - gp1d*  *‘I think when you're unsafe, you have to switch off so many parts of your body, you can't respond in the way that you would normally do. Because of how that might affect your immediate safety. You can't say the thing that you want to say you have to change your personality, you have to change your likes, your interests, your your social circles, maybe you have to change the way in which you socialise and work. So you, you know, and it comes back to the erosion of sense of self that happens with with abuse. But if you're constantly switching off, you’re de-connecting everything in your body of who you are, you know, down to your core. So it is about creating those little connections again, it's like plugging back in to the different, you know, parts of yourself to say, you know, to fully yet be in your body and know, like, well, what can I do now? Like, what can I be? What can I - who am I again? But it is a survival mechanism that we do to, to, yeah, keep yourself safe.’ -dfv2a* |
| **Draw inwards and centre myself too:**  care for self as person, sensor, container, reflector, integrator, healer | *‘Reminds me of interaction ritual theory where you build rapport and trust, and connection through shared emotional experiences… and being able to recognise in ourselves when we are beginning to be uncomfortable’ – P4i*  *‘..when my body could actually feel and process all of the emotions and the physiological impact on it so that I was feeling safe and good.’ – dfv4b*  *‘draw inwards to rely on my own cultural connections so I know who I am and where I am going’ -Ru1k*  *‘keep myself really regulated’ -P3i*  *‘draw on my experience’ - P3i*  *‘Being grounded yourself’ - dfv2g*  *‘know your shit… know who you are and what your stuff is’ - dfv1g*  *‘sense of connectedness even to yourself’ - dfv2g*  *‘you are more valuable than the pills you prescribe’ – gp16a*  *‘But then I also had the same memories of when I had felt distressed, it was almost like there was something at the centre of me that holds that that I know if I can access that can still feel that within me.’ - gp3d*  *‘Or I'll notice that, you know, there's a sort of a tension in my neck, and I'm like, or my body posture slightly shifts, or something inside of me changes in response to the person. So if they go into a zone that I haven't worked on my stuff yet, and I noticed a different response in me, but I would notice a lot of physical sensations in my body that don't feel like mine …so I would discover.’ - gp3d*  *‘In my physical, sort of presence and being with somebody as well as you know, emotionally, spiritually, all of that, but, but I notice it mostly in my body and being to respond in a more or amore being able to stay present, but also being able to maybe be a bit agile and being able to move in and out of how I’m feeling physically while I’m with somebody. So, even if I’m, you know, responding on a physical level to what somebody’s saying, it doesn’t stay with me. So my sense of safety allows me to, to move quite flexibly in how I’m feeling physically when I’m with someone.’ – mhc1e* |
